# Global research trends in atherosclerosis: A bibliometric and visualized study

**DOI:** 10.3389/fcvm.2022.956482

**Published:** 2022-08-23

**Authors:** Wende Tian, Tai Zhang, Xinyi Wang, Jie Zhang, Jianqing Ju, Hao Xu

**Affiliations:** ^1^Xiyuan Hospital, China Academy of Chinese Medical Sciences, Beijing, China; ^2^Graduate School, China Academy of Chinese Medical Sciences, Beijing, China; ^3^National Clinical Research Center for Chinese Medicine Cardiology, Xiyuan Hospital, China Academy of Chinese Medical Sciences, Beijing, China; ^4^Department of Gastroenterology, Xiyuan Hospital, China Academy of Chinese Medical Sciences, Beijing, China; ^5^Graduate School, Beijing University of Chinese Medicine, Beijing, China

**Keywords:** atherosclerosis, inflammation, immunology, bibliometrics, hotspots

## Abstract

**Background:**

Increasing evidence has spurred a considerable evolution of concepts related to atherosclerosis, prompting the need to provide a comprehensive view of the growing literature. By retrieving publications in the Web of Science Core Collection (WoSCC) of Clarivate Analytics, we conducted a bibliometric analysis of the scientific literature on atherosclerosis to describe the research landscape.

**Methods:**

A search was conducted of the WoSCC for articles and reviews serving exclusively as a source of information on atherosclerosis published between 2012 and 2022. Microsoft Excel 2019 was used to chart the annual productivity of research relevant to atherosclerosis. Through CiteSpace and VOSviewer, the most prolific countries or regions, authors, journals, and resource-, intellectual-, and knowledge-sharing in atherosclerosis research, as well as co-citation analysis of references and keywords, were analyzed.

**Results:**

A total of 20,014 publications were retrieved. In terms of publications, the United States remains the most productive country (6,390, 31,93%). The most publications have been contributed by Johns Hopkins Univ (730, 3.65%). ALVARO ALONSO produced the most published works (171, 0.85%). With a betweenness centrality of 0.17, ERIN D MICHOS was the most influential author. The most prolific journal was identified as *Atherosclerosis* (893, 4.46%). *Circulation* received the most co-citations (14,939, 2.79%). Keywords with the ongoing strong citation bursts were “nucleotide-binding oligomerization (NOD), Leucine-rich repeat (LRR)-containing protein (NLRP3) inflammasome,” “short-chain fatty acids (SCFAs),” “exosome,” and “homeostasis,” etc.

**Conclusion:**

The research on atherosclerosis is driven mostly by North America and Europe. Intensive research has focused on the link between inflammation and atherosclerosis, as well as its complications. Specifically, the NLRP3 inflammasome, interleukin-1β, gut microbiota and SCFAs, exosome, long non-coding RNAs, autophagy, and cellular senescence were described to be hot issues in the field.

## Introduction

Atherosclerosis, a chronic disease of arteries and the principle cause of cardiovascular diseases, has now surpassed communicable diseases to become the world’s most prevalent killer (1). Atherosclerosis is triggered by endothelial dysfunction and is associated with retention and modification of low-density lipoproteins (LDL) in the intima (2, 3). The interaction of modified LDL with atherogenic factors promotes the activation of ECs, resulting in the recruitment of monocytes to the intima (4, 5). Differentiated monocytes and vascular smooth muscle cells (VSMCs) avidly capture modified LDL, promoting the formation of foam cells. The inflammatory pathways are also activated, facilitating the formation of fatty streaks, the first sign of atherosclerosis, which is characterized by substantial lipid accumulation within the cells (macrophages and VSMCs) and extracellular space (6). The resulting infiltration of fatty tissue rich in inflammatory leukocytes appears macroscopically as plaques.

Atherosclerosis can occur in any one of many vascular beds; however, coronary heart disease, peripheral artery disease, cerebrovascular disease, or aortic atherosclerosis are examples of atherosclerotic cardiovascular disease (ASCVD). Prolonged pro-inflammatory conditions result in atherosclerotic lesions progressing to an advanced stage, in which macrophage apoptosis increases and the clearance of apoptotic cells decreases (7, 8). It is partly because of this combination that plaque necrosis, a critical feature of vulnerable plaques which contributes to occlusive luminal thrombosis, myocardial infarction (MI), strokes and sudden cardiac death, occurs (9).

As far as atherosclerosis is concerned, clinical strategies focus primarily on relieving symptoms of cardiovascular diseases and preventing future cardiac events (10). As is well known, hypercholesterolemia is one of the major factors in both atherosclerosis initiation and progression (5). Hence, several therapeutic approaches and strategies have been developed to interfere with lipoprotein metabolism as potential therapeutic options (11). As demonstrated by large-scale evidence from the use of 3-hydroxy-3-methylglutaryl-coenzyme A (HMG-Co-A) reductase inhibitors (statins), a 25–50% reduction in the risk of major adverse cardiovascular events (MACEs) can be achieved for each mmol/L lowering in LDL cholesterol (LDL-C) (12). However, it is troubling that despite widespread statin usage, recurrence of MACE continue to be unacceptable among patients with established cardiovascular disease, with 10–12% rates of event after 1 year and 18–20% at 3 years after an index MI (13). It can be attributed in large part to the complex etiology of atherosclerosis, whose pathogenic basis extends much further than intimal cholesterol accumulation (14). In this sense, we must challenge ourselves to deal with the remaining burden of residual risk.

Several opportunities and challenges related to a broad range of topics regarding atherosclerosis prompts a flurry of research into the area, whose enormous volume, heterogeneity, and variable quality renders it difficult to evaluate the scientific impact of the entire scientific literature, and to identify institutions, countries and researchers engaged in exceptional scientific research (15–17).

A bibliometric science mapping and analysis uses manuscript metadata and bibliographic variables as the basis for compiling, organizing, and reviewing published research, and thus allows for fast analysis of large corpora of works (18). This enables us to identify patterns related to authors, journals, countries, and issues under-researched, as well as issues that have already been addressed. In this context, this study seeks to identify, evaluate, and to visualize research published on atherosclerosis in the past decade with respect to qualitative, semi-qualitative, and chronological contexts using validated bibliometric approaches.

## Materials and methods

### Source of the data and search strategy

In the study, the Science Citation Index Expanded of Web of Science Core Collection (WoSCC) of Clarivate Analytics was chosen due to its high efficiency in representing search results. All searches were conducted on the same day, 30 March, 2022. The literature search was completed for identifying atherosclerosis-specific publications with the following search strategy within title in advanced search: “athero*” or “arteriosclero*” or “arteriolosclero*” or “arterial lipoidosis.”

It was decided to restrict the results of the search to articles and reviews containing the search terms in their titles rather than use the “TOPIC” search in Web of Science. It is accurate to perform a title search because it produces a minimal number of false positives (19, 20). The “TOPIC” search retrieves the title, abstract, author keywords, and *KeyWords Plus* for a specific term, which results in the inclusion of a significant number of off-topic publications (21), suggesting that retrieved documents are not necessarily atherosclerosis-only. Particularly, documents that could only be accessed through the *KeyWords Plus* search were excluded since *KeyWords Plus* is an index term derived from frequently occurring words in documents’ references; however, in most cases, they were irrelevant to the literature’s subject matter (22, 23).

The title-only search may, in fact, result in the loss of some documents (false negative); however, the error (false positive) resulting from “TOPIC” is greater (19). Furthermore, the title-only search approach adopted in this study has been validated and applied in previous research (19–21, 24–26).

This was further verified by comparing terms implemented in titles/abstract and titles/author keywords searches to those used in title search scenario, respectively.

In the title/abstract and title-only search scenarios, 45,815 document differences were identified. A manual review of the top cited publications retrieved by Web of Science of these 45,815 documents revealed that the foregoing search terms were mentioned as marginal keywords rather than as essential components of these literature.

As well, 9,846 documents differed between the titles/author keywords and title-only search scenarios; for these 9,846 documents, subsequent review of their top cited publications revealed that they dealt extensively with hypertension, dyslipidemia, heart failure, diabetes mellitus, metabolic syndrome, obesity and chronic kidney disease.

Accordingly, the title/abstract search yielded the greatest number of faulty entries in comparison with the other two search strategies, whereas the titles/author keywords search returned documents that covered an array of diseases with atherosclerosis only one part of the research topics, indicating that these search results were not focused solely on atherosclerosis. This prompted the authors to choose the title search rather than title/abstract or title/author keywords searches, which ensured the highest accuracy and minimum acceptable error.

Atherosclerosis has a changing face. Over the past decade, a combination of fundamental research and clinical investigations has enabled us to radically alter traditional concepts of atherosclerosis; for example, it was previously thought that atherosclerosis mainly affected developed countries; however, the burden of atherosclerosis now falls primarily on developing countries; inflammation is now thought to be related to dyslipidemia and other risk factors, also challenging the long held view that atherosclerosis is a lipid storage disease; plaques exhibiting the classical vulnerable morphology are on the decline in an era of intense lipid lowering, and superficial erosion appears to be on the rise at present (27).

In addition, in the past decade, atherosclerosis research has progressed thanks to advances in human genetics studies enabled by next-generation sequencing and other technological innovations (such as bulk and single-cell RNA sequencing), as well as the ever-evolving toolkit for genetic manipulation of mice (28, 29). In addition to DNA and mRNA analysis, non-coding RNAs have also been studied more closely in the context of atherosclerosis. The transcription of genes implicated in atherosclerosis is altered by microRNAs (miRNAs) and long non-coding RNAs (lncRNAs) (30, 31).

With these advances in understanding the biology of atherosclerosis, therapeutic interventions are likely to be developed that will improve prevention and treatment of this ubiquitous disease. Based on these, we have included the literature in the last decade (from 30 March, 2012 to 30 March, 2022) in this study in order to better present the latest hotspots and the rapidly evolving landscape in this field.

Additionally, we searched the Science Citation Index Expanded for articles and reviews with the search strategy in the title with no limitation of time, yielding 31,120 records. After performing the bibliometric analysis on the entire dataset of 31,120 papers, VOSviewer and CiteSpace were overloaded, thus hindering further visualization of the bibliographic information. As a result of these considerations, the authors chose a period of 10 years as a basis for the study.

Further, the literature search was limited to the English language. Each document’s metadata was compiled and exported in plain text manually. Ultimately, these bibliographic records were imported into CiteSpace and VOSviewer for analysis. The variables used for the analysis were the author, institution, country, journal, reference, and author keyword. The amount of scientific literature published each year was visualized using Microsoft Excel 2019.

### Data analysis and visualization with citespace

As part of the study, CiteSpace was employed to analyze research literature that illustrates the structure of scientific knowledge through the analysis and visualization of data to display various knowledge graphs (32). In CiteSpace, co-citation and co-occurrence networks were visualized, including co-cited references, co-cited journals as well as co-occurrence of authors, institutions, countries, and author keywords, which facilitate the delivery of atherosclerosis knowledge domain results.

Co-citation is the frequency with which two subjects are cited together; it is based on the assumption that co-cited subjects are conceptually related. The co-citation analysis seeks to capture the intellectual structure of the field and may be particularly relevant for systematic reviews, as it reveals how groupings evolve continually and independently of the original publications. An analysis of co-occurrence networks shows how frequently entities occur together.

The co-occurrence analysis illustrates the statistical relationship between two entities within a given dataset; that is, the greater the frequency with which two entities simultaneously occur, the stronger their expected logical connection.

Networks and clusters of co-citation (or co-occurrence) were identified as the primary outcomes of the present study. CiteSpace’s automatic cluster labeling and summarization aid in the interpretation of these clusters.

The parameters of CiteSpace were set as follows: time slicing (2012–2022), years per slice (1), term source (all selection), node type (choose one at a time), and pruning (pathfinder).

**FIGURE 1:**
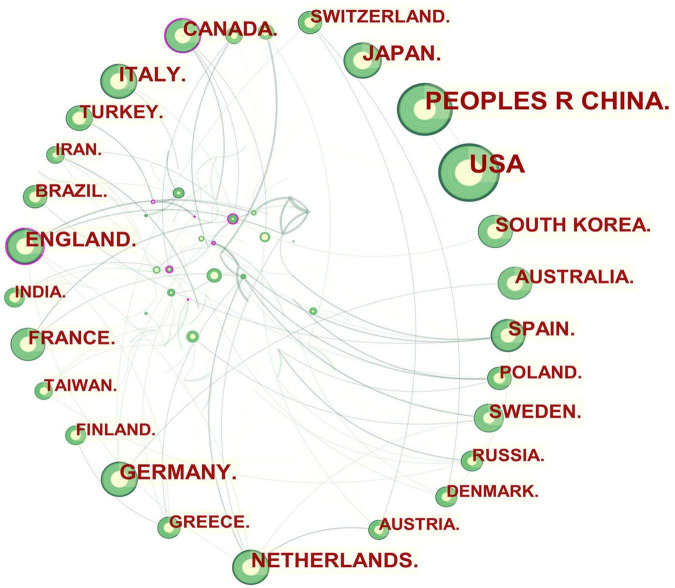
Collaboration network of countries and regions engaged in atherosclerosis research. In the network map, a node represents a country or region. The larger the area of the node is, the larger the number of publications. The thicker the curved line connecting nodes indicates the frequency with which they co-occur, as they indicate collaborative relationships. An isolated node without any connection is devoid of all collaboration. A node with a high betweenness centrality (>0.1) (that is, a node interconnected to more than 10% of the other nodes) exerts substantial influence over others because more information passes through that node. A purple rim also indicates a high degree of betweenness centrality. Red tree rings indicate bursts of citation, i.e., high scholarly activity. The greater the thickness of the red tree rings, the greater the bursts for the corresponding node.

**FIGURE 2:**
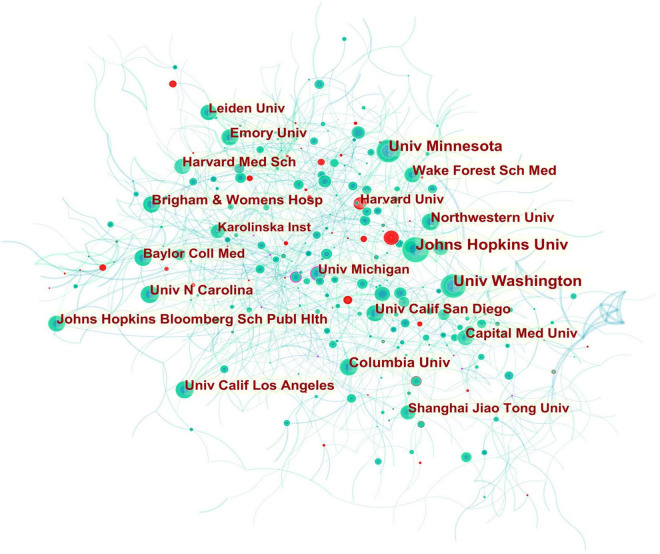
Collaboration network of institutions engaged in atherosclerosis research. In the network map, a node represents an institution. The volume of each node (institution) corresponds to the number of publications, and connecting lines between nodes indicate bidirectional relationships between the institutions; the thickness of the line indicates the strength of the bidirectional collaborative relationships. Isolated institutions lack any collaboration. A node with a high betweenness centrality (>0.1) (that is, a node interconnected to more than 10% of the other nodes) exerts substantial influence over others because more information passes through that node. A purple rim also indicates a high betweenness centrality. Red tree rings indicate bursts of citation, i.e., high scholarly activity. The greater the thickness of the red tree rings, the greater the bursts for the corresponding node.

**FIGURE 3:**
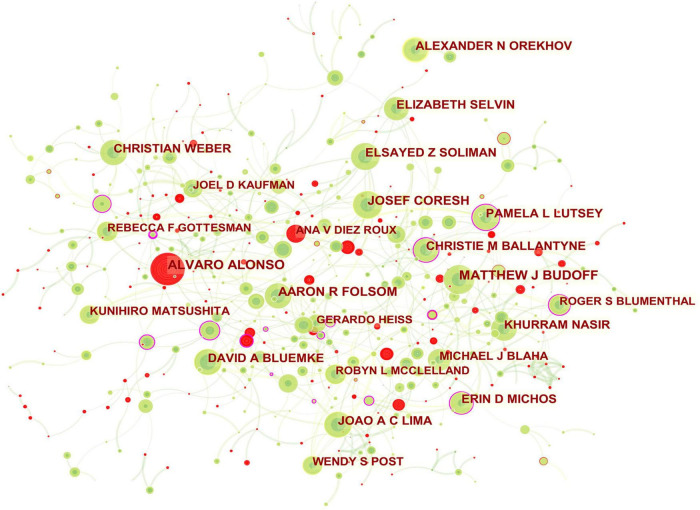
Collaboration network of authors engaged in atherosclerosis research. In the network map, a node represents an author. The volume of each node (author) corresponds to the number of publications, and connecting lines between nodes indicate bidirectional relationships between the authors; the thickness of the line indicates the strength of the bidirectional collaborative relationships. Isolated authors lack any collaboration. A node with a high betweenness centrality (>0.1) (that is, a node interconnected to more than 10% of the other nodes) exerts substantial influence over others because more information passes through that node. A purple rim also indicates a high betweenness centrality. Red tree rings indicate bursts of citation, i.e., high scholarly activity. The greater the thickness of the red tree rings, the greater the bursts for the corresponding node.

There are nodes and links in CiteSpace visualization (Figures 1–3), where nodes represent entities such as authors, organizations, nations, author keywords, journals, and so forth. Depending on the weight of the element, such as the number of publications, citations, or the frequency of occurrence, the size of a node was determined (33, 34). Each node had a color assigned to it, and the same color referred to a cluster, which was a group of entities in the network that shared similar properties. Specifically, a node with a red inner ring is captured with a citation burst, which indicates that the node (e.g., an author, institution, or a country) has been actively publishing for a specific period of time. Relative distance is an approximation of the strength of a relationship between two nodes.

In this way, several key results could be identified, such as entities that were revolutionary in a field (visualized as purple rings), centrality indicators reflecting a entity’s status in the field, citation bursts (hot topics of research), and citation tree rings which represent a node’s year-wise citation pattern (Figures 1–3).

#### Betweenness centrality

The betweenness centrality of a node can be determined when two or more nodes are connected in an area (Eq. 1).


(1) $$C\,e\,n\,t\,r\,a\,l\,i\,t\,y\,\left(n\,o\,d\,e\,i\right)=\sum_{i\neq j\neq k}\frac{\rho \,j\,k\,\left(i\right)}{\rho \,j\,k}$$

In this equation (1), *ρjk(i)* represents the number of paths passing through node *i*, while *ρjk* represents the number of shortest paths between nodes *j* and *k*. Unweighted shortest paths between nodes in a graph are calculated using this algorithm. Each node is assigned a score based on the number of shortest paths that pass through it.

In this way, betweenness centrality can be used to assess the relative importance of each entity (node) within a network; that is having a high betweenness centrality allows for the node to act as a “bridge” between different entities, as it lies on the shortest path between other nodes and connects components of a network that may otherwise be disconnected, if that node was removed (35).

The betweenness centrality score in CiteSpace is normalized to the interval [0, 1]. A node that has a high betweenness centrality (>0.1) is considered to be influential in the network and likely to control significant resources. Or, to put it another way, an entity (such as an author, institution, or a nation) with a high betweenness centrality indicates potentially revolutionary material and its high level of engagement with counterparts. A purple rim is applied to nodes with a high betweenness centrality in CiteSpace. The thickness of the purple rim is proportional to the strength of its centrality betweenness.

#### Citation burst

**FIGURE 4:**
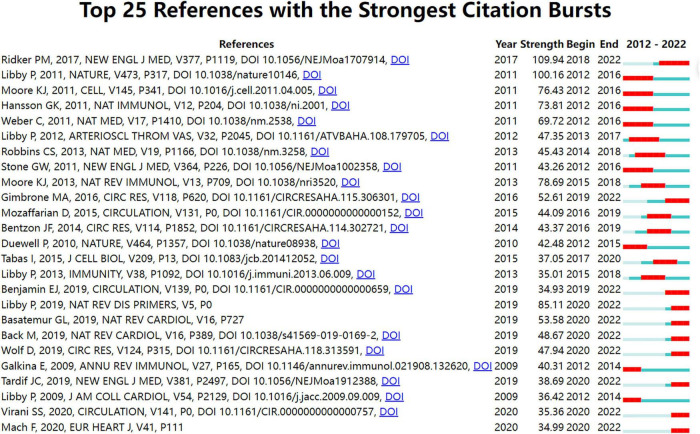
Top 25 references with strong citation bursts in atherosclerosis research. Strength denotes the citation burst strength; the burst strength indicates the rate of change. A citation burst of greater strength is therefore indicative of a period when there has been a sharper surge of citations. A thin blue line marks the entirety of the period between 2012 and 2022; the location and length of the thick red line denotes the time intervals during which reference bursts occur, i.e., rapid increases in citations.

**FIGURE 5:**
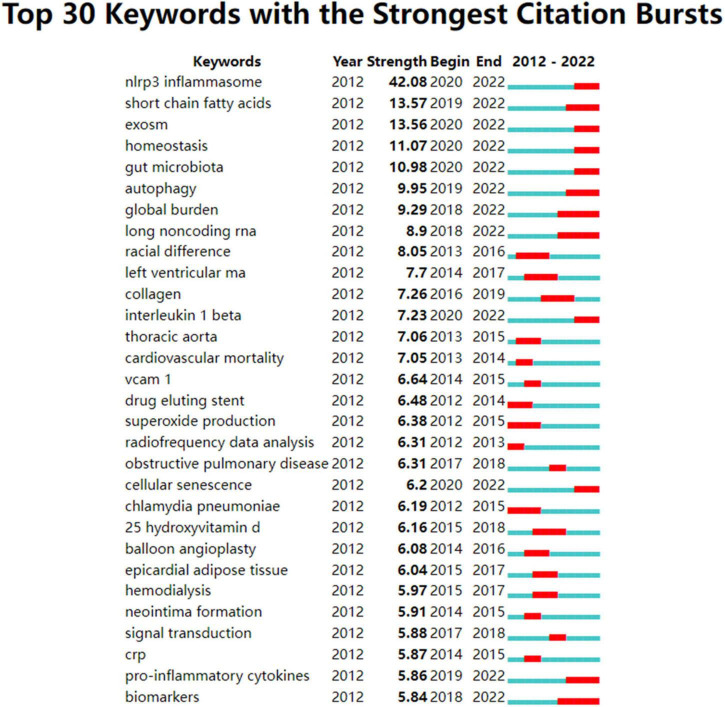
Author keywords with strong citation bursts in atherosclerosis research. Strength denotes the citation burst strength; the burst strength indicates the rate of change. A citation burst of greater strength is therefore indicative of a period when there has been a sharper surge of citations. A thin blue line marks the entirety of the period from 2012 to 2022, whereas red line segments represent the time slices during which keyword bursts occur, i.e., rapid increases in citations.

The citation burst of a node is characterized by a high level of scholarly activity (Figures 1–3) or a rapid increase in citations (Figures 4, 5). Through Kleinberg’s algorithm (36), CiteSpace explores the citation bursts of nodes within a given network. The burst indicator can be detected either for a single node (e.g., an author, author keyword, reference, or a journal, etc.) or for an entire cluster (37).

In terms of an individual node, such as an author keyword, reference, or a journal, a burst is defined by its start year, the end year (and therefore, its duration), and the strength. Strength denotes the citation burst strength. The burst strength indicates the rate of change. A citation burst of greater strength is therefore indicative of a period when there has been a sharper surge of citations, which allows emergent terms to be identified.

Therefore, an indication of the shift in research focus and the duration of the burst can be found in the year the burst began or ended.

In addition, if a cluster includes multiple nodes with strong citation bursts, the cluster as a whole is indicative of an active field of research or an emerging trend.

#### Co-citation

As previously described, in co-citation analysis, it examined how primary publications cite pairs of secondary publications. In specific, it uses co-citation counts, which are defined as the number of times two publications are cited simultaneously (38), to determine semantic similarity (39).

Co-citation analysis, in essence, assumes that publications that are cited together are more intellectually related. The publication co-citation identifies publications that are frequently co-cited, regardless of whether they constitute part of the research field in question. Nevertheless, they can be of extreme importance to the development of the research field. The property helped us identify highly cited publications not included in our database because they were published in a book or journal that was not yet indexed at the time they were published.

The co-citation of two journals is a measure of the frequency at which they are both referenced by a third journal. The high co-citations of two journals are indicative of high semantic relationships between the two; meanwhile, a high co-citation of a journal indicates that it is a prominent source that contains literature relevant to the examined research domain, which have been cited in other articles.

#### Cluster visualization and labeling

A co-occurrence of author keywords provides a glimpse of how the author keywords are interconnected.

Within an examined dataset, author keyword co-occurrence analysis determines the statistical correlation between two author keywords; that is, the more often two terms are mentioned together, the stronger their logical connection is expected to be. This analysis is specifically based on the assumption that co-occurrence of author keywords defines those semantic or conceptual groups of topics able to indicate a field by describing the content of documents. Hence, through algorithmically analyzing author keywords in a set of documents and quantifying their relationship, we are able to determine the extent to which these author keywords are connected. This allows us to construct a conceptual network representation of the research areas.

CiteSpace’s clustering function was used to determine the major entities within the network in which the nodes (i.e., co-occurring author keywords) could be grouped. There are three functions available in CiteSpace to label clusters: Log-Likelihood Ratio, Latent Semantic Indexing, and Mutual Information. Further, cluster labels were automatically extracted using the Log-Likelihood Ratio method. In terms of uniqueness and coverage, this method provided the best results (37). The Latent Semantic Indexing and Mutual Information methods are also available, but were not used in this study because their precision is inferior to that of Log-Likelihood Ratio (37).

**FIGURE 6:**
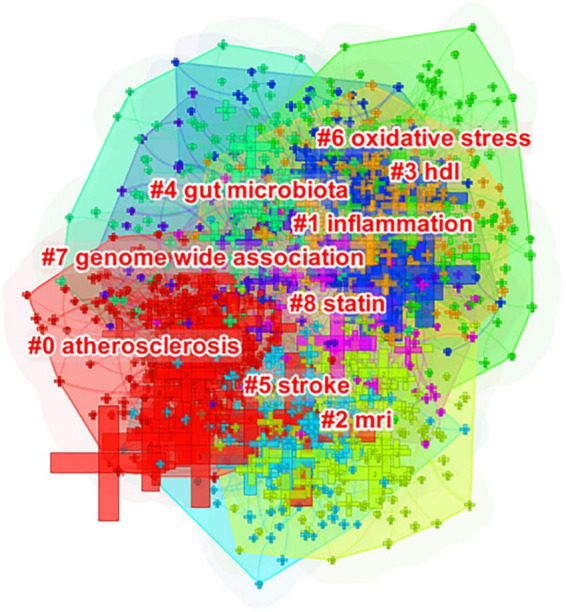
Author keyword clustering knowledge map of atherosclerosis research. Co-occurring author keywords were grouped into different clusters. A cluster is assigned a tag number, and the smaller it is, the more author keywords the cluster contains.

The cluster view and timeline view were subsequently used to analyze the co-occurrence networks. In the cluster view, co-occurring author keywords were grouped into clusters. Co-occurring author keywords are thus referred to as cluster members. On the map, the cluster labels identified by the Log-Likelihood Ratio method illustrate the core topics of each cluster (Figure 6). In detail, clusters are determined by the number of co-occurring author keywords that have a strong connection; thus, clusters are numbered based on their size, commencing with the largest (#0) to the smallest (#8). Using cluster analysis, it is possible to identify major research topics within this knowledge structure.

**FIGURE 7:**
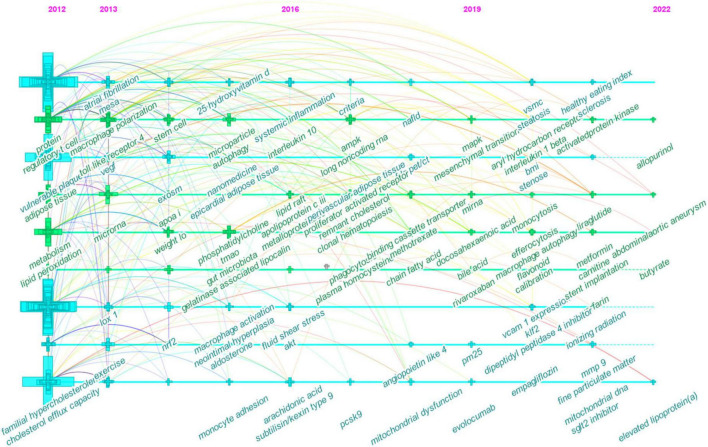
Timeline view of co-occurring author keywords map of atherosclerosis research. Each node represents a author keyword, and the colors represent the average year of publication for each node. The size of a cross corresponds to the citation burst of a keyword co-occurrence.

The timeline view, on the other hand, displayed vertical lines which corresponded to time zones in chronological order from left to right. Nodes (co-occurring author keywords) arranged horizontally are semantically related and belong to the same cluster as indicated by the cluster view. In Figure 7, for instance, the nodes labeled as systemic inflammation, macrophage polarization, adenosine monophosphate-activated protein kinase (AMPK), and mitogen-activated protein kinase (MAPK) on the second line correspond to #1 inflammation (Figure 6). Meanwhile, vertical links can exist between nodes in different time zones; that is, a vertical link between two nodes also indicates that author keywords belonging to different clusters may co-occur.

### Data analysis and visualization with VOSviewer

**FIGURE 8:**
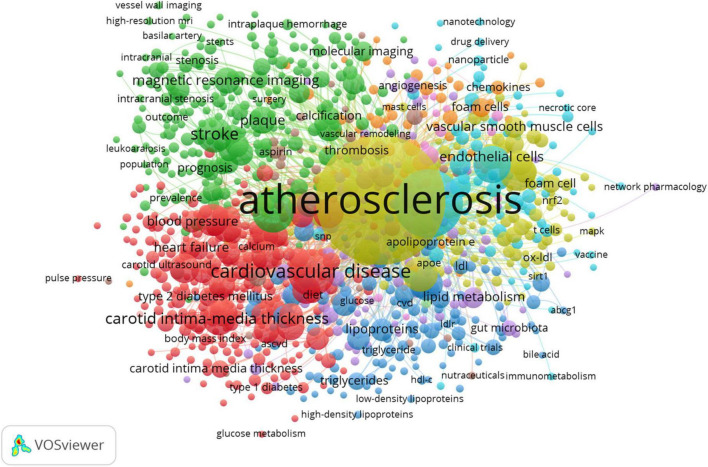
Topic mapping and clustering based on affinities of atherosclerosis research. Minimum number of co-occurrence of a keyword = 10, minimum links strength = 10. There are 5 clusters of author keywords. Mapped author keywords are related to proximity on the map based on author keyword relatedness. Author keyword frequency is represented by the size of the nodes, and the number of documents in which the author keywords occur is illustrated by the weight of connecting lines. Colored clusters indicate where author keywords co-occur (56). Based on the coding principles of grounded theory, including open and axial coding, the cluster names are derived to identify common topics among co-occurring author keywords (56).

To further unpack the co-occurrence network of author keywords, the VOSviewer software (40, 41) was utilized with its filtering capabilities. To reduce oversaturation of a highly used author keyword in the network map, the author keywords of various documents were initially unified according to their forms of writing. For example, “coronary heart disease” and its variations (e.g., “coronary heart disease” and “coronary artery disease”) were merged. Following the exclusion of author keywords that co-occurred less frequently (less than 10 times), the co-occurring author keywords network was created (Figure 8).

Based on the modularity-based clustering method, VOSviewer generates networks that include nodes and links. To elaborate, the nodes represent research entities (author keywords), and links (edges) identify connections between these entities (42). In proportion to how many times a author keyword has been used, the size of the node increases. There is a closer relationship between proximate nodes, and link thickness is related to the strength of the connection between them (how frequently are author keywords co-used). A further feature of VOSviewer is that it categorizes author keywords into separate clusters with different colors, indicating smaller groups of author keywords with strong links among them.

## Results

### Publication output

**FIGURE 9:**
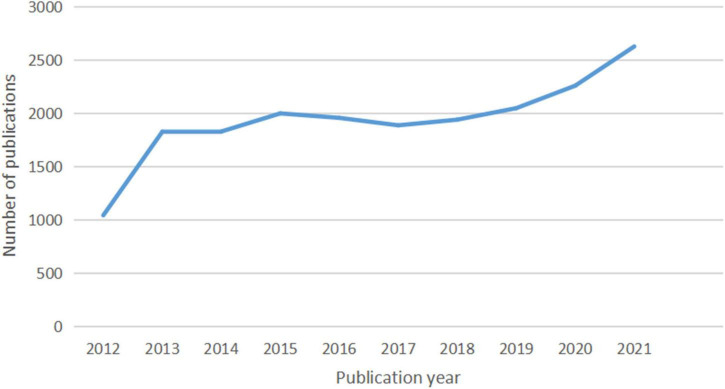
The number of documents published annually in atherosclerosis research.

In total, 20,014 documents, including 17,157 (85.72%) articles and 2,857 (14.28%) reviews, were published. The growth and trends in these documents retrieved from the WoSCC database over the past decade are outlined in Figure 9. From 2012 to 2021, the annual output of studies specifically addressing atherosclerosis went up in three stages.

At the first stage, the quantity of documents has experienced rapid growth from 1,042 to 1,826 papers at the beginning (2012–2013) and a slowdown after the initial explosion from 1,826 to 1,998 (2013–2015). When it comes to the second stage from 2015 to 2017, the annual number of publications exhibited a decreasing trend from 1,998 to 1,886 documents. During the past 5 years, the number of publications increased yearly. Growth was seen in two phases: the first (2017–2019) saw a slow growth from 1,886 to 2,047 publications, and the second (2019–2021) saw a far faster growth from 2,047 to 2,626.

### Countries or regions and institutions analysis

A total of 149 countries or regions participated in the publication of studies on atherosclerosis in the past decade. A table depicting the contribution of each country or region is shown in Table 1. The United States (6,390, 31.93%) was the largest contributor, followed by China (5,028, 25.12%), Japan (1,350, 6.75%), and Germany (1,252, 6.26%).

**TABLE 1:** The top 10 countries or regions and institutions involved in atherosclerosis research.

Rank	Country	Centrality	Count (% of 20,014)	Rank	Institutions	Centrality	Count (% of 20,014)
1	the United States	0.06	6,390 (31,93)	1	Johns Hopkins Univ (the United States)	0.07	730 (3.65)
2	China	0	5,028 (25.12)	2	Univ Washington (the United States)	0.02	675 (3.37)
3	Japan	0	1,350 (6.75)	3	Univ Minnesota (the United States)	0.06	586 (2.93)
4	Germany	0.07	1,252 (6.26)	4	Columbia Univ (the United States)	0.02	375 (1.87)
5	Netherlands	0.08	1,135 (5.67)	5	Harvard Med Sch (the United States)	0.04	362 (1.81)
6	England	0.12	1,118 (5.59)	6	Capital Med Univ (China)	0.03	355 (1.77)
7	Italy	0.07	1,106 (5.53)	7	Univ N Carolina (the United States)	0.05	337 (1.68)
8	Canada	0.11	813 (4.94)	8	Johns Hopkins Bloomberg Sch Publ Hlth (the United States)	0.02	328 (1.64)
9	South Korea	0.01	7,81 (4.06)	9	Univ Calif Los Angeles (the United States)	0.09	318 (1.59)
10	Spain	0.06	7,02 (3.51)	10	Wake Forest Sch Med (the United States)	0.01	307 (1.53)

A total of 921 institutions participated in the atherosclerosis studies. Johns Hopkins Univ accounted for 3.65% of all publications worldwide with 730 publications, the most of any institution. Univ Washington was the second prolific institution with 675 (3.37%) publications, followed by Univ Minnesota with 586 (2.93%) publications, and Columbia Univ with 375 (1.87%).

In CiteSpace, betweenness centrality is used to measure the significance of a theme in the evolution of an entire research field, which recognizes its pivotal points. England (0.12) ranked first by the betweenness centrality, followed by Canada (0.11), and Netherlands (0.08). Univ Calif Los Angeles (0.09) ranked first by the betweenness centrality, followed by Johns Hopkins Univ (0.07), and Univ Minnesota (0.06).

An overview of international research collaborations on atherosclerosis among the participating countries can be seen in Figure 1. England, Canada, and Netherlands, which had a large number of publications involved international scholars. For example, England, which possessed the broadest scientific collaboration, worked intensively with France, Italy, Netherlands, Scotland, Greece, Cuba, South Africa, Kenya, Cyprus, and Pakistan. The main collaborators with Canada were Portugal, Latvia, the United States, Sri Lanka, Israel, Qatar, Cuba, and Kuwait. Netherlands had close cooperation with Germany, Austria, Belgium, Sweden, Ireland, England, Kenya, and Mongolia.

In Figure 2, it is found that most of the institutions that belong to North America were the pivotal points of this collaborating network. Univ Calif Los Angeles cooperated frequently with Univ Calif San Francisco, Univ Chicago, Johns Hopkins Univ, Univ Michigan, Drexel Univ, Cornell Univ, Ronald Reagan UCLA Med Ctr, and Qingdao Univ. Johns Hopkins Univ had close communication with Wake Forest Univ, Wake Forest Sch Med, Univ Minnesota, Johns Hopkins Bloomberg Sch Publ Hlth, Univ Wisconsin, Univ Washington, Tufts Univ, Mayo Clin, Northwestern Univ, and Univ Calif Los Angeles.

In addition, Univ Michigan (0.16) had a high betweenness centrality highlighted in a purple rim, and thus was identified as a key node that linked different countries. Active collaborations were seen among Univ Michigan, Univ Calif San Francisco, Brown Univ, Wayne State Univ, Broad Inst MIT and Harvard, VA Boston Healthcare Syst, Drexel Univ, Univ Calif Berkeley, Univ Calif Los Angeles, Cent South Univ, and Univ Yamanashi. A citation burst was detected for Harvard University, signifying a large increase in recent publications.

### Authors

In total, 733 authors contributed in these documents published in this theme. As shown in Table 2, of the top ten contributing authors, ALVARO ALONSO was ranked first in the number of published articles (171, 0.85%), followed by MATTHEW J BUDOFF (165, 0.82%), JOSEF CORESH (141, 0.70%), and AARON R FOLSOM (135, 0.67%). The top authors by the betweenness centrality were ERIN D MICHOS (0.17), KHURRAM NASIR (0.09), and MATTHEW J BUDOFF (0.07).

**TABLE 2:** The top 10 authors of atherosclerosis research.

Rank	Author	Count (% of 20,014)	Centrality
1	ALVARO ALONSO (the United States)	171 (0.85)	0.06
2	MATTHEW J BUDOFF (the United States)	165 (0.82)	0.07
3	JOSEF CORESH (the United States)	141 (0.70)	0.05
4	AARON R FOLSOM (the United States)	135 (0.67)	0.01
5	DAVID A BLUEMKE (the United States)	131 (0.65)	0.03
6	ELSAYED Z SOLIMAN (the United States)	130 (0.65)	0.02
7	ALEXANDER N OREKHOV (Russia)	128 (0.64)	0
8	ERIN D MICHOS (the United States)	120 (0.60)	0.17
8	ELIZABETH SELVIN (the United States)	120 (0.60)	0.04
9	JOAO A C LIMA (the United States)	119 (0.59)	0.02
10	KHURRAM NASIR (the United States)	112 (0.56)	0.09
10	CHRISTIAN WEBER (Germany)	112 (0.56)	0.05

The scientific collaboration among authors is presented in Figure 3. The network mainly related to American authors. For example, ERIN D MICHOS who had the highest betweenness centrality and played a pivotal role in this domain had close ties with ROBYN L MCCLELLAND (the United States), PAMELA L LUTSEY (the United States), NORRINA B ALLEN (the United States), DI ZHAO (the United States), and OLUSEYE OGUNMOROTI (the United States). Moreover, the leading authors in this field also included CHRISTIE M BALLANTYNE (the United States; 0.12 betweenness centrality), PAMELA L LUTSEY (0.11 betweenness centrality), ROGER S BLUMENTHAL (the United States; 0.11 betweenness centrality).

The main collaborators with CHRISTIE M BALLANTYNE were JOHN J P KASTELEIN (Netherlands), RON C HOOGEVEEN (the United States), VIJAY NAMBI (the United States), and STEVEN E NISSEN (the United States). PAMELA L LUTSEY had frequent communication with FAYE L NORBY (the United States), ALVARO ALONSO, KAMAKSHI LAKSHMINARAYAN (the United States), SUSAN REDLINE (the United States), MARY R ROONEY (the United States), JAMES S PANKOW (the United States), AARON R FOLSOM, and ERIN D MICHOS. ROGER S BLUMENTHAL worked intensively with MICHAEL J BLAHA (the United States), SETH S MARTIN (the United States), SALIM S VIRANI (the United States), KHURRAM NASIR, and LESLEE J SHAW (the United States). ALVARO ALONSO and ANA V DIEZROUX who were captured with citation bursts have actively published in this field recently.

### Journals and co-cited academic journals

Table 3 represents the productive journals and highly co-cited journals in this domain with their number of publications, share of publications, co-citation counts, and impact factor (IF). Seven out of the ten prolific journals have their scopes on cardiovascular disease.

**TABLE 3:** Top 10 journal and top 10 co-cited journals in atherosclerosis research.

Rank	Journal	Count (% of 20,014)	IF	JCR	Rank	Co-cited Journal	Co-citations (% of 536,265)	IF	JCR
1	*Atherosclerosis* (Ireland)	893 (4.46)	5.162	Q1	1	*Circulation* (the United States)	14,939 (2.79)	29.690	Q1
2	*PloS One* (the United States)	605 (3.02)	3.240	Q2	2	*Atherosclerosis* (Ireland)	11,464 (2.14)	5.162	Q1
3	*Arteriosclerosis, Thrombosis, and Vascular Biology* (the United States)	510 (2.55)	8.311	Q1	3	*Arteriosclerosis, Thrombosis, and Vascular Biology* (the United States)	11,377 (2.12)	8.311	Q1
4	*Scientific Reports* (England)	307 (1.53)	4.379	Q1	4	*The New England Journal of Medicine* (the United States)	9,511 (1.77)	91.245	Q1
5	*Journal of the American Heart Association* (England)	292 (1.46)	5.501	Q1	5	*Journal of the American College of Cardiology* (the United States)	9,136 (1.70)	24.094	Q1
6	*International Journal of Molecular Sciences* (Switzerland)	229 (1.14)	5.923	Q1	6	*Circulation Research* (the United States)	8,011 (1.49)	17.367	Q1
7	*Journal of Atherosclerosis and Thrombosis* (Japan)	185 (0.92)	4.928	Q1	7	*PloS One* (the United States)	7,456 (1.39)	3.240	Q2
8	*Circulation Research* (the United States)	177 (0.88)	17.367	Q1	8	*European Heart Journal* (England)	6,428 (1.20)	29.983	Q1
9	*Circulation* (the United States)	174 (0.87)	29.690	Q1	9	*The Journal of Clinical Investigation* (the United States)	6,328 (1.18)	14.808	Q1
10	*International Journal of Cardiology* (Ireland)	171 (0.85)	4.164	Q2	10	*Lancet* (England)	6,190 (1.15)	79.321	Q1

Among them, *Atherosclerosis* had the maximum number of publications (893, 4.46%) with an IF of 5.162, followed by *PloS One* with publications of 605 (3.02%) with an IF of 3.240, and *Arteriosclerosis, Thrombosis, and Vascular Biology* (510, 2.55%) with an IF of 8.311.

The journal co-citation refers to the frequency with which two journals are cited together; this concept is based on the assumption that what is cited together has conceptual affinity. It has been found that *Circulation* with an IF of 29.690 received the highest co-citations (14,939, 2.79%), followed by *Atherosclerosis* (11,464, 2.14%) and *Arteriosclerosis, Thrombosis, and Vascular biology* (11,377, 2.12%). There is a concurrence of *Atherosclerosis*, *PloS One*, *Arteriosclerosis, Thrombosis, and Vascular biology*, *Circulation Research.*, and *Circulation* in the prolific journals and highly co-cited ones.

### Co-cited references and references with citation bursts

Among the 20,014 atherosclerosis documents, there were 1,588 co-cited references. Table 4 shows the top 10 co-cited research publications. Among them, Ridker PM et al. (43) published an article, entitled “*Antiinflammatory Therapy with Canakinumab for Atherosclerotic Disease*” in *The New England Journal of Medicine.*, which was the most co-cited and ranked first (607), followed by “*Progress and challenges in translating the biology of atherosclerosis*,” written by Libby P et al. (44) in *Nature.* (345), “*Macrophages in atherosclerosis: a dynamic balance*,” authored by Moore KJ et al. (45) in *Nature Reviews. Immunology.* (284), and “*Endothelial Cell Dysfunction and the Pathobiology of Atherosclerosis*,” published by Gimbrone MA Jr et al. (46) in *Circulation Research.* (275).

**TABLE 4:** Top 10 co-cited references in atherosclerosis research.

Rank	Reference	Co-citations	Journal	Year
1	Antiinflammatory Therapy with Canakinumab for Atherosclerotic Disease	607	*The New England Journal of Medicine.*	2017
2	Progress and challenges in translating the biology of atherosclerosis	345	*Nature.*	2011
3	Macrophages in atherosclerosis: a dynamic balance	284	*Nature Reviews. Immunology.*	2013
4	Endothelial Cell Dysfunction and the Pathobiology of Atherosclerosis	275	*Circulation Research.*	2016
5	Macrophages in the pathogenesis of atherosclerosis	264	*Cell.*	2011
6	The immune system in atherosclerosis	255	*Nature Immunology.*	2011
7	Atherosclerosis: current pathogenesis and therapeutic options	241	*Nature Medicine.*	2011
8	Atherosclerosis	230	*Nature Reviews. Disease Primers.*	2019
9	Evolocumab and Clinical Outcomes in Patients with Cardiovascular Disease	221	*The New England Journal of Medicine.*	2017
10	Heart disease and stroke statistics—2015 update: a report from the American Heart Association	193	*Circulation.*	2015

Table 5 lists the top co-cited documents based on the betweenness centrality. Of the eleven references, two were published in *Journal of the American College of Cardiology.*, two were published in *The Journal of Clinical Investigation.*, two were published in *Nature Immunology.*, two were published in *European Heart Journal.*, and the other three were from *The New England Journal of Medicine.*, *Lancet.* and *Nature Reviews. Cardiology.*, respectively.

**TABLE 5:** Top 3 co-cited references with the highest betweenness centrality in atherosclerosis research.

Rank	Reference	Centrality	Journal	Year
1	Plaque Characterization by Coronary Computed Tomography Angiography and the Likelihood of Acute Coronary Events in Mid-Term Follow-Up	0.08	*Journal of the American College of Cardiology.*	2015
2	Ezetimibe Added to Statin Therapy after Acute Coronary Syndromes	0.07	*The New England Journal of Medicine.*	2015
2	Relationship of C-reactive protein reduction to cardiovascular event reduction following treatment with canakinumab: a secondary analysis from the CANTOS randomized controlled trial	0.07	*Lancet.*	2018
3	Inflammatory Ly6Chi monocytes and their conversion to M2 macrophages drive atherosclerosis regression	0.06	*The Journal of Clinical Investigation.*	2017
3	CD36 coordinates NLRP3 inflammasome activation by facilitating intracellular nucleation of soluble ligands into particulate ligands in sterile inflammation	0.06	*Nature Immunology.*	2013
3	MicroRNA-33-dependent regulation of macrophage metabolism directs immune cell polarization in atherosclerosis	0.06	*The Journal of Clinical Investigation.*	2015
3	The myth of the “vulnerable plaque”: transitioning from a focus on individual lesions to atherosclerotic disease burden for coronary artery disease risk assessment	0.06	*Journal of the American College of Cardiology.*	2015
3	Pathophysiology of native coronary, vein graft, and in-stent atherosclerosis	0.06	*Nature Reviews. Cardiology.*	2016
3	The neuroimmune guidance cue netrin-1 promotes atherosclerosis by inhibiting the emigration of macrophages from plaques	0.06	*Nature Immunology.*	2012
3	The effect of interleukin-1 receptor antagonist therapy on markers of inflammation in non-ST elevation acute coronary syndromes: the MRC-ILA Heart Study	0.06	*European Heart Journal.*	2015
3	Residual inflammatory risk: addressing the obverse side of the atherosclerosis prevention coin	0.06	*European Heart Journal.*	2016

In Figure 4, strong citation bursts for 25 references are shown. The strongest citation burst was the article entitled “*Antiinflammatory Therapy with Canakinumab for Atherosclerotic Disease*” published in *The New England Journal of Medicine.* by Ridker PM et al. (43) with a citation burst lasting from 2018 to 2022 (109.94), followed by “*Progress and challenges in translating the biology of atherosclerosis*” published by Libby P et al. (44) in *Nature.* with a citation burst spanning from 2012 to 2016 (100.16), and “*Macrophages in the pathogenesis of atherosclerosis*,” published in *Cell.* by Moore KJ et al. (6), which showed a citation burst from 2012 to 2016 (76.43).

When focusing on the last 5 years, the analysis of ongoing citation bursts revealed that the topics of the ten references deserve special consideration (43, 46–54).

### Author keywords analysis

In the present study, author keywords were extracted from 20,014 publications. Upon excluding irrelevant keywords and merging those that shared the same semantic meaning, 1,266 author keywords were identified. These keywords were used for constructing a co-occurring author keyword map using the VOSviewer software (55, 56). Mapped author keywords are related to proximity on the map based on keyword relatedness. Figure 8 shows a author keyword co-occurrence network for topics related to atherosclerosis research. These divided into five clusters: lipids and lipoproteins in the development and progression of atherosclerosis (dark blue cluster), the molecular mechanisms and signaling pathways in atherosclerotic plaque cells (yellow cluster), nanocarriers for atherosclerosis treatment (light blue cluster), special features of atherosclerotic plaques (purple cluster), assessment and management of atherosclerosis and its complications (green cluster), and risk factors for atherosclerosis and its thrombotic complications (red cluster).

As can be seen in Figure 6, co-occurring keywords were grouped into different clusters in Citespace. The following nine clusters were presented: #0 atherosclerosis; #1 inflammation; #2 magnetic resonance imaging; #3 high-density lipoprotein; #4 gut microbiota; #5 stroke; #6 oxidative stress; #7 genome-wide association; and #8 statin.

The timeline visualization of co-occurring keywords network is shown in Figure 7. Through this timeline view, key areas of research may be identified to guide the future research orientation.

In the early years from 2012 to 2016, the field began to focus on (1) familial hypercholesterolemia; (2) Multi-Ethnic Study of Atherosclerosis; (3) exercise and weight loss; (4) nanomedicine; (5) epicardial adipose tissue; (6) apo A-I, 25-hydroxyvitamin D, phosphatidylcholine, and aldosterone; (7) gut microbiota and trimethylamine N-oxide (TMAO); (8) vulnerable plaque and neointima formation; (9) cholesterol efflux capacity and lipid peroxidation; (10) regulatory T cells (T_*reg*_) and stem cells; (11) macrophage polarization, macrophage activation, monocyte adhesion, and autophagy; (12) microRNA (miRNA), exosome, and microparticles; (13) nuclear factor erythroid 2-related factor 2; (14) toll-like receptor 4 (TLR4) and lectin-like oxidized LDL (oxLDL) receptor-1; (15) vascular endothelial growth factor and neutrophil gelatinase-associated lipocalin.

In the mid-term phase, from 2016 to 2019, researchers began to focus efforts on (1) non-alcoholic fatty liver disease; (2) [^18^F]-fluorodeoxyglucose (FDG)-positron emission tomography (PET)/computed tomography (CT); (3) methotrexate; (4) systemic inflammation and clonal hematopoiesis; (5) fluid shear stress; (6) lipid raft and remnant cholesterol; (7) short-chain fatty acids (SCFAs); (8) apolipoprotein C-III, arachidonic acid, proprotein convertase subtilisin/kexin type 9 (PCSK9), and plasma homocysteine; (9) matrix metalloproteinase (MMP); (10) phagocytosis and mitochondrial dysfunction; (11) lncRNAs; (12) peroxisome proliferator-activated receptor, adenosine triphosphate-binding cassette protein A1, angiopoietin-like protein 4, protein kinase B (Akt), and AMPK; (13) interleukin-10 (IL-10).

From 2019 to 2022, the field turned to research on (1) abdominal aortic aneurysm, hepatic steatosis, monocytosis, and systemic sclerosis; (2) body mass index; (3) ionizing radiation; (4) rivaroxaban, evolocumab, empagliflozin, allopurinol, liraglutide, metformin, dipeptidyl peptidase-4 inhibitors, and sodium-glucose cotransporter-2 inhibitors; (5) particulate matter (less than 2.5 μm in diameter; PM2.5); (6) lipoprotein(a); (7) bile acid; (8) docosahexaenoic acid, flavonoids, L-carnitine, and butyrate; (9) endothelial to mesenchymal transition, efferocytosis, and macrophage autophagy; (10) VSMCs; (11) aryl hydrocarbon receptor, vascular adhesion molecule-1, Kruppel-like Factor 2, and MAPK; and (12) IL-1β.

Table 6 details the meaningful author keywords with high frequency in this field. Usually, high-frequency author keywords are the primary focus of a research field. The most frequent author keywords included coronary heart disease (3,477), inflammation (2,532), apolipoprotein E (ApoE)-deficient mice (2,165), intima media thickness (2,120), endothelial dysfunction (1,977), oxLDL (1,793), and MI (1,670).

**TABLE 6:** Top 20 author keywords with the highest count in atherosclerosis research.

Rank	Keywords	Count	Rank	Keywords	Count
1	coronary heart disease	3,477	11	all-cause mortality	1,282
2	inflammation	2,532	12	macrophage	1,208
3	ApoE-deficient mice	2,165	13	ischemic stroke	1,106
4	intima media thickness	2,120	14	vascular oxidative stress	9,82
5	endothelial dysfunction	1,977	15	hypertension	860
6	oxLDL	1,793	16	insulin resistance	852
7	MI	1,670	17	prevalence	806
8	cholesterol efflux capacity	1,669	18	C-reactive protein (CRP)	787
9	vulnerable plaque	1,319	19	coronary artery calcification	683
10	vascular smooth muscle	1,284	20	nuclear factor kappa B (NF-κB)	682

Detection of keywords that experienced an influx of appearances or citations over a defined period of time was carried out using a citation burst analysis. As shown in Figure 5, the results revealed that the top keywords ranked by the strength of citation bursts were “NLRP3 inflammasome” (42.08), “SCFAs” (13.57), “exosome” (13.56), “homeostasis” (11.07), “gut microbiota” (10.98), “autophagy” (9.96), etc.

## Discussion

### General information

Research activity can be gauged by the number of publications in a field (57, 58). Figure 9 indicates that the number of publications on this topic has substantially increased over the last decade. The publication of academic papers have more than doubled over this time. It is remarkable that by growing at 10 to 16% per year, the growth rate for scientific production highly increased from 2019 to 2021. As this performance indicates, there have been some sub-fields of interest about the topic over the recent years.

Table 1 shows that countries in North America (the United States and Canada), Asia (China, Japan, and South Korea), and Europe (Germany, Netherlands, England, Italy, and Spain) were the leading driving force in atherosclerosis research. A source’s greater betweenness centrality is directly connected to its greater influence on the subject under examination. As a result, as well as being the prolific countries, England and Canada were leaders in terms of centrality indicator, as indicated in Figure 1. Their global reach is indicative of their influence on atherosclerosis research as well as their high level of engagement with other nations.

With regard to the high-yield institutions, the United States dominated the publication output given that most of contributing institutions in atherosclerosis-related work were located in the United States, except for Capital Med Univ in China. However, the low betweenness centrality pointed out their less academic influence and poor global collaboration. As shown in Figure 2, collaborations for these institutions tended to be intra-country phenomena. It should be noted that the publication of atherosclerosis papers of Univ Michigan (245) failed to make it in the top ten rankings, but its highest betweenness centrality indicated publications from this university greatly influenced research in this decade and its collaboration relationships has more globally diversified.

From the authors’ contribution shown in Table 2, the American researchers kept the leading role in producing publications in the field. However, similar to the research landscape of productive institutions, the low betweenness centrality of these high-yield researchers was indicative of less influence on each other and globally weak collaboration relationships. Instead, Figure 3 pictures a dominance of some authors (e.g., ERIN D MICHOS; CHRISTIE M BALLANTYNE; PAMELA L LUTSEY; ROGER S BLUMENTHAL) over others in atherosclerosis research overall and the central roles in their collaboration community.

Table 3 illustrates the dominance of Western journals in the scientific publication of atherosclerosis research. Studies with well-designed methodologies and high quality are the basis for atherosclerosis domain, as highly prolific journals are typically found in Q1 and Q2. It is through journal co-citation analysis that researchers can gain insight into mainstream journals and their impact. A similar pattern emerged with the most highly co-cited journals being those published in Western countries which are categorized as Q1 or Q2.

These findings, coupled with results obtained from micro (individual scholars), meso (institutions) and macro (nations) levels, emphasize the need for strengthening journal capacity and enhancing global collaboration for Asian countries, therefore enabling high-quality scientific output and disseminating knowledge in the area of atherosclerosis.

Moreover, *Atherosclerosis*, *PloS One*, *Arteriosclerosis, Thrombosis, and Vascular Biology*, *Circulation Research.*, and *Circulation* were considered core journals in the field because of their high publications and co-citations.

### Knowledge base

Based on the analysis of the most co-cited documents, the base literature of research can be identified.

As shown in Table 4, most of the highly co-cited literature were reviews outlining the crucial role inflammation plays in driving atherosclerosis from disease onset through clinical complications and immune cells and vascular cells as the key players in plaque inception and progression.

Other documents included were landmark studies in this field. For example, compared to placebo, canakinumab led to significant reductions in recurrent cardiovascular events, independent of lipid-level lowering, as an anti-inflammatory therapy targeting the IL-1β innate immunity pathway (43). According to the other study, evolocumab, the PCSK9 inhibitor, can lower LDL cholesterol by as much as 60% and reduce the risk of cardiovascular events among patients with established cardiovascular disease (59). Another was an update on statistics regarding heart disease, stroke, and other cardiovascular and metabolic diseases (60).

In Table 4, the top 3 co-cited references with the highest betweenness centrality were considered key in defining the intellectual base of atherosclerosis. For example, Motoyama S et al. (61) demonstrated that patients with progression of high-risk plaques in coronary computed tomography angiography were at 26.7% risk of cardiovascular events; those without plaque progression and without high-risk plaques were at 0.3% risk of acute coronary syndrome. The Improved Reduction of Outcomes: Vytorin Efficacy International Trial has revealed that the addition of ezetimibe, a non-statin drug that inhibits the intestinal absorption of cholesterol by targeting Niemann-Pick C1-Like 1 (NPC1L1), to simvastatin resulted in the lowering of LDL cholesterol levels by approximately 24% and improved the outcome of the patients with coronary artery disease (62). In a secondary analysis of the Canakinumab Anti-inflammatory Thrombosis Outcome Study (CANTOS) trial, it was found that patients who achieved the highest reduction in high sensitivity CRP as a result of canakinumab treatment had better cardiovascular disease outcomes (63). Rahman and colleagues found that Ly6C*^high^* monocyte influx is a prerequisite for plaque regression and differentiation of reparatory macrophages (64). The study by Sheedy FJ et al. (65) has shown that oxLDL priming is dependent on the binding of oxLDL to CD36 and the formation of the CD36-TLR4-TLR6 complex, and after internalization of oxLDL, NLRP3 becomes activated in response to damage to lysosomes. In the Medical Royal Council InterLeukin-1 Antagonist heart study, patients with acute non-ST-elevation MI were randomized to receive either anakinra or a matching placebo daily for 14 days. A decrease in IL-6 and CRP was observed following anakinra administration; however, major adverse cardiac events at 1 year were higher in the anakinra arm than in the placebo arm (66).

The key findings of other articles included (1) macrophage *Aldh1a2*, a gene involved in the metabolism of retinoic acid, was depressed with anti-miR-33 treatment, resulting in the activation of T_*reg*_ and protection against atherosclerosis (67); and (2) netrin-1 as a neuronal guidance cue that mediates chemorepulsion and chemoreattraction of axons through receptor UNC5b, promotes atherosclerotic plaque progression through the retention of macrophages within inflamed blood vessels (68). Others are excellent reviews which highlighted (1) the residual risk of atherosclerotic cardiovascular disease (69); (2) morphological and structural characteristics of atherosclerotic plaques for native coronary disease, vein grafts, and stents (70); (3) the shift from focusing on individual lesions to assessing coronary artery disease risk by the atherosclerotic disease burden based on the fact that affected patients with more vulnerable coronary plaque are more likely to have MACEs, but rarely, the plaques indicated as vulnerable are the cause of acute arterial thrombosis (71).

### Hot topics

In Figure 4, the ten references which had ongoing strongest citation bursts characterized the emerging topics of this field. These papers with their key findings or conclusions are summarized in Supplementary Table 1 to provide an overview (43, 46–54, 72).

Based on the findings previously discussed, it is not difficult to make a conclusion that the field of atherosclerosis is presently engaged in intense research regarding inflammation. Corroborative findings from Figures 5, 6, 8 are a good illustration of this point.

In addition, the keywords whose citation bursts last until 2022 in Figure 5 were identified for exploring the hot themes. Mechanisms implicated in initiation and progression of atherosclerosis were the leading research focus. Among them, NLRP3 inflammasome with downstream factor IL-1β, exosome, gut microbiota with SCFAs, autophagy, lncRNAs, cellular senescence are potential hotspots. Since these topics identified are not separated, but influential and interrelated to each other, to better illuminate these issues and make them more focused, we highlight their interrelated aspects.

(1) NLRP3 inflammasome: In recent studies, atherosclerosis has been recognized as an inflammatory disease associated with lipids, and the NLRP3 inflammasome is implicated in the link between lipid metabolism and inflammation, since NLRP3 inflammasome activation is dependent upon crystalline cholesterol and oxLDL in atherosclerosis plaques (73).

The NLRP3 inflammasome has been associated with atherosclerosis in many studies by analyzing aortic NLRP3 expression in patients with atherosclerosis. NLRP3, caspase-1, and apoptosis associated speck-like protein (ASC) are the key components of the NLRP3 inflammasome, which have been found to be highly expressed in plaques of the aorta and carotid arteries, as well as the subcutaneous fat of patients with atherosclerosis (74, 75). Several studies have also suggested that smoking, hypertension, high sugar intakes, and fatty diets rich in saturated fats may all be responsible for enhanced activation of NLRP3 in myeloid cells of the patients with atherosclerosis (76). Likewise, Paramel Varghese G et al. (74) analyzed the transcripts of the NLRP3 inflammasome and IL-1β in the atherosclerotic plaques of individuals with and without MI. On a transcriptional level, atherosclerotic plaques exhibit dramatic up-regulation of NLRP3, ASC, caspase-1, IL-1β, and IL-18. Also, NLRP3 mRNA levels were significantly increased in plaques of symptomatic patients. The dysregulation of NLRP3 inflammasomes, and particularly its genetic variations, contribute to atherosclerosis (74).

Extensive animal studies have also investigated the role and mechanism of the NLRP3 inflammasome in atherosclerosis. Studies conducted in the early 2000s examined the effects of IL-1β and IL-18, downstream cytokines of the NLRP3 inflammasome, on atherosclerosis in mice. The absence of *Il-1β*, in mice lacking both *ApoE* and *Il-1β*, reduced the size of aortic atherosclerotic lesions, possibly through increased levels of vascular cell adhesion protein 1 (VCAM-1) and monocyte chemotactic protein 1 (77). By inhibiting IL-18 endogenously in mice, early lesion development was prevented and a more stable plaque phenotype was produced with reduced macrophages, T cells, cell death, and lipid content and increased VSMCs and collagen content (78).

Nearly 10 years later, the results of a study by Latz E and colleagues found that inflammasome contributed to the progression of atherosclerosis in mice lacking *Ldlr*; *Nlrp3* or *Il-1*-deficient mice did not suffer from atherosclerosis or systemic inflammatory cytokine responses induced by diet (79, 80). *Nlrp3^–/–^*, *Asc^–/–^* or *Il-1α/β^–/–^* bone marrow transplantation reduced early atherosclerosis simultaneously with a decrease in IL-1β and IL-18 levels in *Ldlr^–/–^* mice (79). In addition, in *ApoE^–/–^* mice treated with the selective NLRP3 inhibitor MCC950 or silencing *Nlrp3* by lentivirus, atherosclerosis progression was reduced, confirming NLRP3 inflammasome as a causative factor (81). Further studies indicate that the NLRP3 inflammasome is not only involved in early atherosclerosis but also in exacerbating vulnerable plaque formation (82).

However, the relevance of the NLRP3 inflammasome in atherogenesis has been disputed in some studies. For example, Menu P et al. (83) found no differences in the progression of atherosclerosis, plaque infiltration, or plaque stability in ApoE-deficient mice that lack either *Nlrp3*, *Asc*, or *caspase-1* compared with wildtype mice, presenting conflicting evidence that atherosclerosis develops independently of NLRP3 inflammasome in *ApoE^–/–^* mice. One explanation of this is the fact that based on the mouse model (*ApoE^–/–^*), IL-1α is primarily responsible for atherosclerosis. The production of IL-1α is not reliant on NLRP3 activation, so lack of its key components would not affect atherosclerosis. In addition, a number of experimental factors could be involved, such as the mouse model, gender, age, and the type of atherogenic diet, along with the high-fat diet feeding time (84), as in female *Ldlr^–/–^* mice, *Nlrp3* deficiency in bone marrow cells is associated with decreased atherosclerosis but not in male mice (85). Further, *ApoE^–/–^* mice used in this study (83) exhibit markedly greater levels of diet-induced atherosclerosis compared to *Ldlr^–/–^* mice used by Latz E et al. (79). The mice also received an atherogenic diet containing more than 8 times as much cholesterol for 11 weeks, which was 3 weeks longer than the previous study (79). In the presence of excessive dietary cholesterol and an extended feeding period, the NLRP3 inflammasome may play a weaker role in the development of atherosclerosis. NLRP3 inflammasome’s role will need to be clarified by further studies.

A majority of studies have shown that monocytes promote VSMC phenotypic switch through activation of NLRP3 inflammasome, which can adversely affect plaque stability (86). There are many mechanisms that can be involved in the activation of the inflammasome, including lysosomal rupture, enhanced potassium ion efflux, mitochondrial dysfunction, endoplasmic reticulum stress, and reactive oxygen species (ROS) release and all are present in plaques, especially in necrotic cores, and yet few studies have been conducted on them in atherosclerosis (87).

A significant decline in MACEs was observed after treatment with canakinumab in CANTOS trial (43). Additionally, the study showed that NLRP3-mediated inflammatory pathways play a critical role in atherosclerosis progression, placing the possibility of specific NLRP3 inhibitors as promising therapies to combat atherosclerosis. Presently, there are two strategies for inhibiting the NLRP3 inflammasome, directly inhibiting NLRP3 or indirectly inhibiting the signaling events downstream. There have been several small-molecule drugs identified that target NLRP3 inflammasome so far, and they are being investigated in preclinical studies of cardiovascular inflammation (88). It is worth noting that specificity of the potent target sites is a prerequisite for developing new inhibitors of NLRP3 inflammasome that can be used therapeutically. Moreover, the challenge remains to optimize the net benefit of these interventions, since interference with other inflammatory pathways may impair host defenses. Hence, whether targeting IL-6, downstream of IL-1 and IL-18, might quell inflammation with less impairment of host defenses, especially since IL-6 is causally involved in atherosclerosis, as shown by Mendelian randomization analysis (89), is an ongoing issue that warrants further investigation.

Referring to IL-1β, the downstream cytokine of the NLRP3 inflammasome, whose multiple effects are observed in all stages of atherosclerosis, it initiates an inflammatory response in ECs by triggering the expression of adhesion factors and chemokines and allowing inflammatory cells to accumulate in blood vessels and penetrate the intima, which is associated with initiation of inflammation in atherosclerosis (90). These adhesion molecules include VCAM-1 and intracellular adhesion molecule-1, and chemokines include monocyte chemoattractant proteins.

Aside from stimulating VSMC proliferation and differentiation, IL-1 also activates monocytes and macrophages and helps release inflammatory mediators (91). Among them, the production of IL-6 and MMP can be induced by IL-1β (92–94). As part of the acute phase response, IL-6 increases CRP, fibrinogen, and plasminogen activator inhibitor levels, which are closely related to atherosclerosis (92–94). Plaque destabilization and rupture is closely linked to MMP-1, MMP-8, and MMP-13 due to their characteristic of degradation of the fibrous cap (95).

The phagocytosis of oxLDL induces the expression of pro-IL-1β and ROS by the cathepsin B pathway, resulting in activation of the NLRP3 inflammasome, inducing macrophages to secrete IL-1β, and promoting macrophage transfer into foam cells during atherosclerosis (96). As a consequence of negative feedback, IL-1β inhibits cholesterol efflux, causing intracellular cholesterol to accumulate and foam cells to form (97).

Consequently, a possible solution to atherosclerosis might be inhibiting IL-1β’s signal transduction, including IL-1 receptor antagonists, the type 2 IL-1 receptor, and soluble receptors (98). In addition to these, there are animal experiments and clinical trials showing that drugs target IL-1β in the treatment of atherosclerosis, including Anakinra, monoclonal antibodies, vaccines, and rilonacept (99).

(2) Exosomes and lncRNAs: Exosomes regulate atherosclerosis with effect on ECs, VSMCs, and macrophages. Through exosomes, donor cells can communicate with recipient cells *via* cargoes such as non-coding RNAs and proteins. As lncRNAs were identified as a trendy topic, here, we focused on exosomes transferring lncRNAs for atherosclerosis progression regulation to present a more specific description of a hot theme.

This topic has been explored in several studies, analyzing exosomes derived from atherogenic cells induced by oxLDL or those from atherogenic patient plasma. We present here a summary of the diversity of donor cells with recipient cells and the lncRNAs that are transported *via* exosomes in regulation of atherosclerosis depicted in Supplementary Figure 1.

The study by Wang Y et al. (100) examined the levels of exosomes and exosomal HIF 1 alpha-antisense RNA 1 (HIF1A-AS1) in 36 patients with atherosclerosis and 28 healthy adults. It was concluded that atherosclerosis patients had significantly elevated levels of exosomes and the exosomal HIF1A-AS1. There is evidence to suggest that exosomal lncRNAs play a role in atherosclerosis diagnosis through their differential expression.

It is proposed that ECs, VSMCs, and immune cells communicate *via* exosomal lncRNAs, which target miRNAs or directly regulate gene expression, to regulate the occurrence and development of atherosclerosis. A study by Chen L et al. (101) found that the expression of lncRNA growth arrest-specific 5 (GAS5) in exosomes derived from THP-1 cells stimulated with oxLDL was significantly upregulated. The increase in apoptosis of ECs following the uptake of exosomes from THP-1 cells expressing high GAS5 levels indicated that exosomal GAS5 stimulates macrophage and EC apoptosis. As a consequence, inhibition of GAS5 may be a useful strategy to treat atherosclerosis. The study of Liang W et al. (102) showed that in patients with atherosclerosis and ECs treated with oxLDL, GAS5 expression was elevated, while miRNA-26a expression was reduced. As a result of GAS5 binding to miRNA-26a, ECs undergo apoptosis and autophagy function is impaired in human aortic ECs. In a study by Zhong X et al. (103), miR-26a-5p ameliorated oxLDL-induced ECs apoptosis by inactivating the TLR4/NF-κB signaling pathway. As a result, in the presence of oxLDL, GAS5 in exosomes from THP-1 cells causes EC apoptosis through down-regulation of miRNA-26a-5p, activation of the TLR4/NF-κB pathway, and up-regulation of apoptotic factors such as caspases.

In a study by Huang C et al. (104), lncRNA metastasis-associated lung adenocarcinoma transcript 1 (MALAT1) was significantly enriched in exosomes secreted by oxLDL-stimulated human umbilical vein endothelial cells (HUVECs) and co-culture with these exosomes enhanced THP-1 cell MALAT1 expression and promoted M2 macrophage polarization. In addition, an up-regulation of MALAT1 expression was observed in exosomes produced by HUVECs treated with oxLDL (101). In human neutrophils treated with exosomes from oxLDL-treated HUVECs, MALAT1-induced exosomal signaling activates the P38/Akt signaling pathway, which leads to the formation of neutrophil extracellular traps (NETs) (101). A murine model of atherosclerosis exposed to exosomes from oxLDL-treated HUVECs developed hyperlipidemia, inflammation, and NETs, suggesting that atherosclerosis exacerbations might occur (105).

The lncRNA retinal non-coding RNA3 (RNCR3) is found to be significantly up-regulated in both human and mouse atherosclerotic lesions in the aorta, accelerating endothelial protection from atherosclerosis (106). The knockdown of RNCR3 in mice models (*ApoE^–/–^* and C57BL/6J) results in aggravated hypercholesterolemia and excessive release of inflammatory factors that enhance atherosclerosis development (106).

LINC01005 is highly expressed in HUVECs treated with oxLDL, and as a result, its expression is also high in the derived exosomes. This lncRNA promotes VSMC proliferation and migration (107). By sponging miR-128-3p, which targets KLF4, LINC01005 also regulates gene expression for a synthetic phenotype. It is demonstrated that by co-culturing exosomal LINC01005 derived from oxLDL-treated HUVECs with VSMCs, the contractile markers α-SMA and SM22a were down-regulated, whereas the VSMC proliferation marker OPN was significantly increased (107–109). As a result, the synthetic phenotype of VSMCs is induced, and therefore atherosclerosis develops.

A study *in vitro* suggests that exosome-mediated lncRNA ZEB1 antisense 1 (ZEB1-AS1) plays an important role in atherogenesis of HUVECs (110). Exosomal lncRNA ZEB1-AS1 promotes cell injury by the miR-590-5p/ETS1 axis in oxLDL-stimulated HUVECs *via* the TGF-β/Smad pathway (110).

(3) Gut microbiota and SCFAs: SCFAs are essential for intestinal health because they act as mediators between the gut, the diet, and the host. This means that they play a critical role in a variety of metabolic processes, including lipid synthesis, fat storage, glucose transport, and inflammation (111). In the colon, dietary fiber and resistant starch are fermented and converted into SCFAs such as butyrate, acetate, and propionate, which comprise more than 90% of all SCFAs (111). Along with the microflora present in the colon, diet, environmental conditions, including pH, as well as the gut transit all contribute to their production (112). Evidence has grown to suggest SCFAs may be related to atherosclerosis. Yet, the effects of different SCFAs on atherosclerosis differ from one another.

The incorporation of acetate into fatty acids and cholesterol in rat hepatocytes is inhibited by propionate, causing a decrease in cholesterol serum levels (113). Further, in two different hypertensive cardiovascular damage mouse models, propionate treatment markedly reduced hypertension, vascular inflammation and atherosclerosis, and cardiac damage. The ability of propionate to modulate immune homeostasis, particularly T_*reg*_ function, was critical for this effect (114).

Since acetate, the most abundant SCFA in peripheral circulation, is a substrate for cholesterol, it encourages cholesterol production (113). The acetate to propionate ratio may therefore decrease serum lipids, which could play a role in reducing cardiovascular risk.

There is also ample evidence that butyrate modulates a variety of atherosclerotic processes. As an early sign of atherosclerosis, endothelial injury, monocyte adhesion, and chemotaxis typically occur due to multiple risk factors. NF-κB is activated and endothelial inflammation is caused either by oxLDL or proinflammatory factors, which cause phosphorylation of IκB or the formation of P65-P50 heterodimers in the nucleus. Butyrate inhibits dimer formation by down-regulating p65 and by preventing its movement into the nucleus. As such, it interferes with the action of NF-κB, which also improves the stability of atherosclerotic plaque (115–117). When consumed orally, butyrate decreases the level of oxLDL in obese patients and decreases the inflammatory response of circulating monocytes (118). Moreover, butyrate inhibits the overproduction of adhesion molecules such as VCAM-1 and E-selectin, thereby preventing monocytes from adhering to injured endothelium (119). The production of ROS and various inflammatory factors is directly regulated by butyrate in the atherosclerosis process. For example, by reducing NADPH oxidase expression and inducible nitric oxide synthase, butyrate has been found to relieve oxidative stress and reduce endothelial dysfunction (120). Together, these studies confirm butyrate’s role as an anti-inflammatory agent and antioxidant stress atheroprotector.

With its regulating function in lipid metabolism, the inhibition of NPC1L1 and the up-regulation of ABC subfamily G member 5 and member 8 transporters were shown to suppress cholesterol uptake in a dose-dependent manner by butyrate (121). Butyrate is responsible for accelerating reverse cholesterol transport and mitigating the formation of atherosclerotic plaques by promoting the expression of ATP-binding cassette subfamily A member 1 and subsequent cholesterol efflux through a specificity protein 1 pathway as demonstrated in mice model lacking *ApoE* induced by high-fat diet (122).

Altogether, an increasing body of evidence indicates that SCFAs may have an impact on atherosclerosis. As one of these agents, butyrate alleviates atherosclerosis alongside efforts to decrease the formation of atherosclerotic plaque, inhibit inflammation, and improve oxidative stress in atherosclerotic lesions, providing insight into a possible therapeutic target. Yet, further studies that provide solid evidence are necessary to move from animal studies to human ones.

(4) Autophagy: Autophagy, also known as macroautophagy, maintains cell homeostasis by releasing unneeded proteins and organelles that can be more efficiently used in a cell’s survival. ECs, VSMCs, and macrophages undergo autophagy when stimulated with pro-atherogenic factors. A cytoprotective effect of basal or moderate autophagy is seen in atherosclerosis; the opposite is true when excessive or dysfunctional autophagy adversely impacts cell survival, leading to atherosclerosis (123). Atherogenesis is mediated by lncRNAs by controlling the autophagy status of plaque cells (ECs, VSMCs, and macrophages). Supplementary Figure 2 illustrates LncRNAs that regulate autophagy in atherogenesis and the mechanisms underpinning these regulatory processes.

With regard to lncRNA-modulated EC autophagy in atherosclerosis, it is reported that GAS5 knockdown reduces cell apoptosis in human aortic ECs in response to oxLDL, decreases SQSTM1/p62 levels, and increases LC3-II/I ratio, and these effects are reversed by suppressing miR-26a expression (102). This suggests that EC apoptosis is exacerbated by impaired autophagy and may be responsible for GAS5’s pro-atherogenic effects. In HUVECs, overexpression of MALAT1 results in increased levels of LC3-II protein and facilitates autophagosome and autolysosome formation by inhibiting the phosphoinositide 3-kinase (PI3K)/AKT pathway (124). Down-regulation of MALAT1 in brain microvascular ECs results in a significant reduction of LC3-II expression and an increase in SQSTM1/p62 levels by targeting the miR-200c-3p/sirtuin 1 pathway (125). Induction of autophagy by MALAT1 in ECs is likely to inhibit inflammation and atherogenesis.

Vascular smooth muscle cells transfected with siMALAT1 displayed heightened expression of contraction-related genes, including α-SMA, SM-22, myocardin, and serum response factor. Further, knockdown of MALAT1 inhibits proliferation and migration in VSMCs. MALAT1 is responsible, in part, for modulating VSMC phenotypes by sponging miR-142-3p, which targets ATG7 and enhances the contractile phenotype (126). Therefore, MALAT1 might have the potential, in addition to its beneficial role in atherogenesis (127), to accelerate atherosclerosis *via* autophagy stimulation, in which it inhibits the contractile phenotype of VSMCs. Moreover, in VSMCs, overexpression of BRAF-activated non-protein coding RNA (BANCR) significantly increases the LC3-II/I ratio and promotes cell proliferation, and the JNK inhibitor SP600125 blocks these effects (128). Further, treatment with the inhibitor of autophagy, 3-MA, significantly diminishes BANCR’s positive effects on autophagy and proliferation of cells (129). By promoting the autophagy and proliferation of VSMCs, BANCR might have atherogenic effects.

According to Li Y et al. (130), THP-1 macrophages stimulated with oxLDL exhibited elevated expression of DYNLRB2-2, a lncRNA that stimulates cholesterol efflux and inhibits foam cell formation by activating autophagy. The miR-298/sirtuin 3 axis was modulated by DYNLRB2-2, resulting in the LKB1/AMPK/mTOR pathway-mediated autophagy in macrophages. This mechanism corroborates earlier observation that autophagy is a key mechanism behind cholesteryl esters reverse transport to lysosomes and subsequent ABCA1 transporter-mediated efflux (131). Another study has demonstrated that DYNLRB2-2 increases ABCA1 expression in THP-1 macrophages stimulated with oxLDL *via* G protein-coupled receptor 119 (GPR119) (132). In support of this, *in vitro* overexpression of GRP119 leads to an increased cholesterol efflux, inhibition of foam cell formation, and activation of a proinflammatory genetic program. Additionally, *in vivo* viral overexpression of GRP119 in *ApoE^–/–^* mice fed high-fat diets shows that it has a protective effect against atherosclerosis by increasing cholesterol efflux and reducing the expression of proinflammatory cytokines (132). These findings suggest that DYNLRB2-2 is a potential candidate for reducing atherosclerotic plaque formation and enhancing cholesterol homeostasis. Additional experiments are needed to determine whether DYNLRB2-2 promotes macrophage autophagy in order to protect against atherosclerosis.

(5) Cellular senescence: Atherosclerosis, an age-related disease, is associated with cellular senescence as well as other physiological processes (133). It has been demonstrated that cellular senescence in the vasculature, referred to as “vascular senescence,” contributes to the pathogenesis of this disease. Cellular senescence is a stable cell cycle arrest in which proliferating cells lose their receptivity to stimuli that encourage growth, generally as a result of DNA damage, which can take two forms - telomere-dependent or intrinsic/replicative senescence and telomere-independent or extrinsic/stress-induced senescence (134). An arrest in the cell cycle heralds the onset of senescence and is characterized by an increase in a number of molecules, including cell cycle regulators such as p16INK4A, p21 and p53, as well as senescence-associated β-galactosidase (SA-β-gal), which is only present in senescent cells (135).

A characteristic of senescent cells is the acquisition of a proinflammatory phenotype known as the senescence-associated secretory phenotype (SASP). Among the components of this secretome are pro-inflammatory cytokines, such as IL-1α, IL-1β, IL-6, IL-8, IL-18, chemokine (C-C motif) ligand 2, and TNF-α (136); growth factors, including TGF-β, vascular endothelial growth factor, and platelet-derived growth factor-AA (137); proteases, such as MMPs (e.g., MMP-1, MMP-3, MMP-8, MMP-9, and MMP-13); extracellular matrix components, such as fibronectin (138); ROS (139); and miRNAs located within exosomes (140). In a mechanical sense, on the one hand, this phenotype may serve as an essential trigger for an efficient immune response that regulates cellular senescence. Meanwhile, SASP may play a major role in turning cellular senescence into an age-related disease pathology (141).

In the absence of an effective immune response, senescent cells are not sufficiently eliminated, which leads to their accumulation (142). The SASP activity may cause damage to the surrounding tissue, resulting in the extension of the senescence process to other tissues and cells, a phenomenon known as the senescence-induced bystander effect (141). Further, cellular senescence stimulates autocrine and paracrine responses, which affect immune-competent cells as well as distant structures.

Studies have demonstrated that senescent cells accumulate in atherosclerotic lesions, both in experimental models and in human plaques, indicating this disease is sculpted by cell senescence (143). Similarly, studies conducted in mice lacking the LDL receptor demonstrated that removing p16INK4a-positive senescent cells from atherosclerotic plaques suppressed typical pathological changes (144).

In atherosclerosis, ECs, VSMCs, macrophages, and other cell types are involved in senescence. In brief, senescent cells play a sequential role in atherogenesis: first, accumulation of senescent ECs initiates plaque formation, which promotes monocyte entry into the vessel through the activation of SASP. Senescent ECs are also more prone to apoptosis, resulting in increased endothelial permeability that facilitates the extravasation of oxLDL. Increasing senescent EC accumulation results in impaired signaling, such as a decrease in nitric oxide secretion, which contribute to early intimal thickening, one of the major risk factors for atherosclerosis. The SASP subsequently mediates plaque progression and destabilization, having pro-atherosclerotic effects. As a result, the senescent cells contribute to the destabilization of the plaque, which in turn is more prone to rupture, which can lead to acute complications such as strokes and MI.

Specifically, senescent cells have a dual negative impact on atherosclerosis. In the first instance, the buildup of senescent cells within atherosclerotic lesions results in cell dysfunction as well as impede tissue repair. Second, in senescent cells, a complex secretome, previously referred to as the SASP, is actively produced. SASP elements are liberated when senescent cells accumulate, causing low-grade inflammation to persist. By perpetuating the release of proinflammatory factors, caused by the accumulation of senescent cells, inflammation can become a chronic condition, contributing to plaque vulnerability.

Loss of functional physiological activity of ECs is connected with cellular aging and is regarded as a pathogenic mechanism in the early stages of endothelial damage and atherosclerosis. Specifically, on one hand, it has been demonstrated that senescent ECs produce reduced nitric oxide and exhibit increased expression of the adhesion molecules vascular cell adhesion molecule 1 (VCAM1) and intercellular adhesion molecule 1 (ICAM1), which bind monocytes to induce endothelial infiltration (145). The senescence of ECs directly destroys the endothelial barrier by interfering with cell proliferation, permeability, and motility, thereby causing endothelial erosion and intraplaque hemorrhage. *In vitro* data suggest that senescent ECs are more susceptible to apoptosis and possess compromised tight junction formation, which may elevate oxLDL retention in the arterial intima and lead to atherogenesis (146, 147). In addition, at the bifurcation sites of vessels, where blood flow is disrupted, ECs display shorter telomeres, which indicates an exacerbation of senescence.

On the other hand, by stimulating monocyte recruitment and inflammatory responses, the SASP effect of senescent endothelial cells leads to the progression of plaque vulnerability. The activation of p53/p21 signaling results in reduced migration and altered expression of inflammatory factors in senescent ECs induced by disturbed flow (148). In addition, it is also possible for senescent cells to develop the SASP and to produce increased levels of both soluble factors and extracellular vesicles that serve as carriers of senescence signals (149). Atherosclerosis and thrombosis may be exacerbated by senescent ECs that produce increased levels of IL-1, IL-6, IL-8, IL-15, monocyte chemoattractant protein-1 (MCP-1), TNF, and other mediators (150).

As a result of DNA damage, extracellular vesicle secretion is significantly increased in most cases; this impairs the ability of ECs to regenerate, thus decreasing their potential for cell migration and vascular formation (151). As part of the SASP, the EC-derived microvesicles may facilitate the release of insoluble proteins and activate specific signaling pathways in target cells (152), possibly contributing to the development of atherosclerotic plaque. For example, the calcification of human aortic SMCs has also been reported to be stimulated by microvesicles from elderly individuals’ plasma or senescent ECs (153). Besides the microvesicles in plasma being more abundant with age, senescent EC-secreted microvesicles also contain higher quantities of calcium and calcium-binding proteins, which are involved in vascular calcification, suggesting that microvesicles may serve as markers of vascular calcification in atherosclerotic plaques (153).

In this regard, a potential antiatherogenic effect may be achieved by regulating senescent ECs. A study by Hayashi T et al. (154) revealed that T0901317, an agonist of liver X receptor, inhibited atherosclerosis and specifically EC senescence in a rat model of diabetic atherosclerosis, partly by inducing endothelial nitric oxide synthase and inhibiting ROS. The knockdown of *angptl2* promoted endothelial repair and limited the progression of atherosclerotic lesions in aorta walls by eliminating endothelial senescent cells (155). It was also suggested by Kheloufi M et al. (156) that an sufficient level of endothelial autophagy protected ECs against inflammation, senescence, apoptosis, and atherosclerosis development.

Atherosclerosis involves the presence of senescent VSMCs primarily in the intima rather than in the mesenchyme (157), and the senescence of VSMCs is related more to plaque size than to the plaque formation (158). The proliferation of VSMCs that produce extracellular matrix facilitates the stabilization of plaques and the fibrous cap of atherosclerosis (159). Senescent VSMCs, however, secrete matrix-degrading proteases, which can contribute to the vulnerability of plaques. The collagen secretion from senescent VSMCs is reduced compared with normal VSMCs, further impairing the stability of plaques (160). Thus, senescent VSMCs are not only found in atherosclerosis, but their properties also aggravate its development and increase the risk of complications associated with atherosclerosis.

Oxidative stress has been demonstrated to accelerate telomere shortening because telomeres are rich in guanine and consequently susceptible to oxidation to 8-Oxoguanine (8oxoG) (161). Atherosclerosis severity has been strongly associated with telomere shortening, and telomere length has been implicated as a putative risk factor for ASCVD (162, 163). It was found that human fibrous cap VSMCs have significantly shorter telomeres than normal VSMCs (164). Senescence of VSMCs in atherosclerosis may be induced not only by a decrease in telomere length, but also by the loss of telomere-binding factors and structural alterations to telomeres (165). It has been demonstrated that the loss of telomeric repeat-binding factor-2, which is crucial for maintaining telomeres, promotes plaque VSMC senescence and exacerbates plaque instability in atherosclerosis (166).

Aside from these, VSMC senescence in atherosclerotic mice is also reported to be induced by DNA damage (167). Physiological or pathological ROS are the most likely agents responsible for DNA damage, and it is this guanine’s lower redox potential that makes the base particularly susceptible to oxidative damage (165). Shah A et al. (168) demonstrated that 8oxoG basal excision repair (BER) defects exist in VSMCs of human atherosclerotic plaques due to decreased expression and acetylation of 8oxoG DNA glycosylase (OGG1). As evidenced by BER-deficient mice’s severe premature aging and metabolic deficiencies, BER is an essential component of genome integrity and maintenance (169).

In addition to the decreased proliferative capacity of senescent VSMCs in the fibrous cap, which may result in an instability of the atherosclerotic plaque, other studies suggest that VSMC senescence contributes to plaque destabilization *via* stimulation of inflammation. An array of SASP factors are secreted by senescent human VSMCs, including IL-6, IL-8, and MCP-1, which are mediated by IL-1α; anti-inflammatory factors, such as CC chemokine ligand and IL-1R2, are however, reduced (170). Specifically, senescent human VSMCs are capable of actively promoting the development of atherosclerosis and plaque rupture through the release of SASP (170). As a result of the autocrine stimulation of senescent VSMCs by IL-1α, the SASP generates sustained secretions of a variety of inflammatory factors and chemokines (170). In response to these potent chemotactic signals, monocytes and lymphocytes aggregate, while released IL-1α activates neighboring normal VSMCs and ECs, further resulting in pro-inflammatory cytokines secretion and increased adhesion receptor expression (170).

Overall, senescent VSMCs can induce persistent inflammation associated with atherosclerosis by exhibiting an IL-1α-driven SASP and inducing a pre-atherosclerotic condition in neighboring cells (170). Atherosclerosis may be targeted by blocking IL-1α, a potential source of chronic inflammation (170). Moreover, it has been shown sirtuin protein 6 delayed VSMC senescence by preserving telomere integrity, thus reducing the burden of atherosclerotic plaques and promoting their stability (171). There is evidence that the inhibitor of dipeptidyl peptidase-4, alogliptin, is protective against the senescence induced by IL-1β in VSMCs (172). Therefore, therapies targeting the reduction of inflammation may provide therapeutic benefits in the form of anti-atherosclerosis agents that promote plaque stability by delaying VSMC senescence.

In addition, a pro-calcification phenotype is observed in aging VSMCs in response to inflammation and oxidative stress and multiple osteogenic pathways including runt-related transcription factor 2 (Runx2), bone morphogenetic protein-2 (BMP-2), alkaline phosphatase, osteopontin, and osteoprotegerin are activated (173, 174). It is possible, therefore, that VSMCs may take on an osteoblast-like phenotype, thereby increasing susceptibility to calcification, which is associated with cardiovascular complications and may also contribute to plaque vulnerability (174–176).

Furthermore, in atherosclerosis, VSMC autophagy, senescence, and apoptosis are interrelated and negatively associated (160). In VSMCs, autophagy is moderated by a balance of oxLDL, with low to moderate concentrations promoting autophagy and elevated levels inhibiting it (177). When moderately activated, autophagy removes unnecessary or dysfunctional components and protects VSMCs against senescence (178–180). In contrast, inhibition of autophagy promotes the senescence of VSMCs. By activating LKB1/AMPK/mTOR signaling dose- and time-dependently, genistein protected VSMCs from aging by inducing autophagy (181). Further, a recent study found that nifedipine restored senescence-impaired autophagic activity, which could prevent hydrogen peroxide-induced senescence in VSMCs *via* modulating SA-β-gal activity and the expression of p53, p21, and senescence marker protein 30 (182). Moreover, in the presence of anti-apoptotic proteins, senescent VSMCs are characterized as anti-apoptotic (183). Autophagy is inhibited during apoptosis to ensure complete cell death. Thus, in the setting of atherosclerosis, VSMC autophagy, senescence, and apoptosis are all interconnected. The imbalance among these pathways may be responsible for the development of unstable atherosclerotic plaques (160).

Senescent immune cells found in the vasculature wall contribute to atheroma development as well. When compared to age-matched controls, T cells with shortened telomeres were more pronounced in coronary heart disease patients than myeloid cells, implying that T cells play a significant role in aging and atherosclerosis (184). In several studies, senescent leukocytes have been identified as contributing to atherosclerotic plaque progression and senescent effector memory T (T_*EMRA*_) cells are present in unstable plaques (185). An analysis of leukocyte populations indicates that telomere shortening is a predictor of atherosclerosis and cardiovascular disease; moreover, both CD4^+^ and CD8^+^ T_*EMRA*_ cells are considered to predict cardiovascular-related mortality in older individuals (186–188). Aside from the pro-inflammatory phenotype, T_*EMRA*_ cells exhibit multiple cellular senescence traits, including decreased proliferation, mitochondrial failure, increased production of TNF and IFN-γ, and increased p38 MAPK activation (189). In addition, T_*EMRA*_ cells exhibit atypical cytotoxic activity toward plaque ECs, potentially resulting in plaque erosion (173). The accumulating CD8^+^ CD28*^null^* CD27^–^ senescent T cells on the inflammatory cardiovascular wall continuously produce IFN-γ, which triggers macrophages to release MMPs for extracellular matrix destruction (190–192). This is a key underlying mechanism of T cell-related atherosclerosis pathogenesis.

Moreover, patients with advanced atherosclerosis have been found to exhibit monocytes that generate high levels of ROS and pro-inflammatory cytokines (144, 193). Cellular senescence is responsible for these pro-inflammatory phenotypic changes in macrophages (194). A histological examination of the early atherosclerotic lesion using transmission electron microscopy revealed a large buildup of foamy macrophages in the fatty-streak lesions, which were accompanied by intact elastic fibers and no fibrous cap (144). However, sub-endothelial senescent foamy macrophages may produce VCAM1 and MCP-1 to recruit circulating monocytes, which accelerated the growth of senescent foamy macrophages and resulted in the production of various inflammatory cytokines and MMPs (e.g., MMP-3 and MMP-13) (144). At the late stage of atherosclerosis, senescent foam cells in atherosclerosis-prone *Ldlr^–/–^* mice promoted elastic fiber degradation, fibrous cap thinning, and plaque instability (144).

It has been reported that the treatment of mouse macrophages and human peripheral blood mononuclear cells with lipopolysaccharide for 24 h promoted the senescence through the NF-κB-induced activation of bromodomain-containing protein 4 (BRD4)-dependent epigenetic way (195). Senescent macrophages had a clear SASP condition as well as increased lipid uptake, accelerating the progression of atherosclerosis (195). The suppression of BRD4 with inhibitors such as siBRD4, JQ-1, or I-BET762 prevented the senescence of macrophages and the accumulation of lipids by reducing the expression of the SASP proteins (195). As a result of this study, we can gain a deeper understanding of immunosenescence and make informed decisions about future drug research.

As indicated in the analysis, the forgoing topics represent hot issues in atherosclerosis field. As it turns out, these molecular targets or biological processes are interrelated in a complex pattern that have mutually affecting effects. Here, we provide an updated analysis of some of them intertwining.

(1) Interplay between NLRP3 inflammasomes and intestinal microecology: There is increasing evidence that the NLRP3 inflammasome and gut microbiota are emerging as important links that affect atherosclerosis formation and development. It was reported that NLRP3-deficient mice demonstrated reduced inflammation, decreased bile acids, and different expression patterns of fatty acids (196–198). Changes in gut microbiota composition occurred in conjunction with these changes, which are related to reduced levels of systemic TMAO and lipopolysaccharides (196–198). Furthermore, the gut microbiota and its metabolites modulate NLRP3 inflammasome activation, and gut dysbiosis is worsened by NLRP3 activation (199, 200). Several studies have demonstrated that the NLRP3 inflammasome and gut microbiota are also involved in the occurrence and development of atherosclerosis *via* regulating lipid metabolism, inflammation, oxidative stress, and other mechanisms (201, 202). Here, we chose to focus on the interaction between gut microbiota with its metabolites (e.g., TMAO and SCFAs) and NLRP3 inflammasome in atherosclerosis.

The mechanism by which TMAO activates NLRP3 inflammasome has been extensively investigated in recent years, which involves in oxidative stress aggravation and, ultimately, endothelial dysfunction. Importantly, a recent study has identified TMAO as a novel and independent risk factor promoting atherosclerosis through the induction of vascular inflammation. In HUVECs as well as the aortas of *ApoE^–/–^* mice, it was also demonstrated that TMAO activated inflammation *via* the NOD-like receptor family and may trigger the activation of the NLRP3 inflammasome *via* the sirtuin 3 (SIRT3)-superoxide dismutase2 (SOD2)-mitochondrial ROS (mtROS) signaling pathway (203). In the study by Sun X et al. (204), by triggering oxidative stress and activating the thioredoxin interacting protein (TXNIP)-NLRP3 inflammasome, TMAO induced increased production of IL-1β and IL-18 in a dose- and time-dependent manner. Collectively, TMAO can stimulate ROS oxygen radicals *via* indirect pathways and activate TXNIP-NLRP3 and SIRT3-mtROS in mitochondria, thereby stimulating the synthesis of the inflammatory cytokines IL-1, IL-18, and caspase-1. Apart from these, TMAO was also shown to enhance the secretion of NLRP3 in inflammasome, aggravating endothelial injury by directly activating p38-MAPK and NF-κB signaling pathways (205, 206).

Vascular calcification is the result of disseminated mineral deposition inside the medial layer of arteries. The steps of osteogenic differentiation, matrix maturation, and matrix mineralization are used to characterize it as an active osteogenic process of vascular cells, mostly VSMCs. Zhang X et al. (206) demonstrated that TMAO promoted calcium/phosphate-induced calcium deposition in VSMCs of rats dose-dependently, and promoted expression of bone-related molecules such as Runx2 and BMP2. The osteogenic differentiation of VSMCs was suggested to be facilitated by TMAO (206). TMAO activated the NF-κB/NLRP3/caspase-1/IL-1β signaling during this process (206). Therefore, TMAO may promote vascular calcification through activation of these elements.

The effects of SCFA, an inhibitor of histone deacetylase, have been shown in Caco-2 tumor cells that have been stimulated with LPS to suppress expression of all NLRP3 components, attenuate intestinal barrier dysfunction, inhibit ROS generation, and activate autophagy (207). The results of a study using a partial ligated carotid artery mouse model indicated that butyrate reduced cholesterol-induced activation of NLRP3 inflammasomes within arterial walls (208). Butyrate was thought to play a protective role by inhibiting the lipid raft redox signaling pathway and decreasing cholesterol crystal- and 7-ketocholesterol-mediated free radical generation (208). In addition, the inhibitory effects of acetate on NLRP3 inflammasome activation are modulated by the G-protein-coupled receptor 43 and Ca^2+^-dependent mechanisms, which provides further relevance to the mechanism of attenuation of atherosclerosis development by metabolites regulating NLRP3 inflammasome activity (209).

Also, TLR8-dependent activation of the NLRP3 inflammasome by bacterial RNA has recently been demonstrated in human myeloid cells (210). Intestinal *salmonella* and *proteus* were also shown to cause mitochondrial dysfunction through endoplasmic reticulum stress, release ROS, and trigger the activation of the NLRP3 inflammasome (211). It is yet to be determined whether these interactions between gut microbiota and NLRP3 inflammasome activation also directly regulate lipid metabolism, inflammation, oxidative stress, and endothelial dysfunction in the setting of atherosclerosis.

The aforementioned research shed light on microbiota dysbiosis (or gut microbiota-derived metabolites)-mediated NLRP3 inflammasome activation in atherosclerosis, supporting the possibility of development of treatment options including gut microbiota composition manipulation in combating atherosclerosis. The NLRP3 inhibitor (MCC950), a caspase-1 inhibitor (YVAD), as well as NLRP3 short interfering RNA were shown to reduce TMAO-mediated NLRP3 inflammasome activation, thereby inhibiting inflammation in HUVECs (203). It has also been shown that treatment with MCC950 restored the abundance and composition of the gut microbiota to that of normal mice in experimental autoimmune encephalomyelitis mice (212). Further, as evidence mounts, NLRP3 inflammasome and their downstream mediators have emerged as important targets for statin drugs in inflammatory diseases (213). A study in mice treated with atorvastatin or rosuvastatin showed that the genera *Bacteroides*, *Butyricimonas*, and *Mucispirillum* were enriched (214). Rosuvastatin was found to be more effective in restoring the altered gut microbiota caused by a high fat diet (214). Accordingly, the therapeutic potential offered by these agents by targeting NLRP3 inflammasome seems to be partly dependent on modulating gut microbiota and microbial metabolites. A further investigation is warranted, however, since this association has not been confirmed.

Some natural molecules, such as resveratrol (215), berberine (216, 217), and ferulic acid (218), have been shown to influence the gut microbiota and bile acids, fatty acids, lipids, and glycolytic metabolites, while affecting the assembly and activation of the NLRP3 inflammasome, thereby protecting against atherosclerosis. Among the other strategies, trimethylamine (TMA) lyase inhibitors (219), probiotics (220), fecal microbial transplantation (221) may also be employed to restructure the gut microbiota or inhibit TMA/TMAO, which directly or indirectly regulate NLRP3 inflammasome activation. In addition, SCFAs may be leveraged as an emerging therapy for atherosclerosis *via* regulation of NLRP3 inflammasome activity (222).

It is noteworthy, however, that although there is increasing interest in the effect of intestinal flora and metabolic pathways on NLRP3 inflammasome activation, research regarding their relation to atherosclerosis is still in its infancy. Taking SCFAs as an example, the safety and effectiveness of exogenous supplemental SCFAs, as well as their pharmacokinetics and pharmacodynamics, the precise mechanisms between the SCFAs at various concentrations and NLRP3 inflammasome activation in atherosclerosis, and the dearth of well-designed and controlled human intervention studies, to name a few, still remain open questions.

(2) Anti-atherosclerosis *via* exosome targeting cellular senescence: SASP is a mechanism for cell-to-cell communication during senescence, as was described above. Increasingly comprehensive quantitative proteomics analyses are revealing soluble and exosomal components of the SASP, some of which are abundant in the plasma of humans during senescence and age-related diseases. Furthermore, exosomes were recently suggested to be critical mediators of the SASP’s paracrine senescence action (223). For example, a role for exosomes in interorgan long-distance communication is demonstrated in non-alcoholic fatty liver disease and cardiovascular disease models (224). In their study, Jiang F et al. (224) demonstrated that hepatocyte-derived miR-1, loaded into and transferred by exosomes, accelerated endothelial inflammation and facilitated atherogenesis through suppressing KLF4 and activating NF-κB. Age-related exosomes significantly increase vascular calcification, which is a risk factor for atherosclerosis and cardiac damage. An increase in annexin A6, BMP2, and Ca^2+^ levels was observed in exosomes isolated from replicative senescent ECs and blood plasma of elderly patients, which were found to initiate and propagate calcification in human aortic smooth muscle cells (153, 225). In contrast, the prothrombin-mediated inhibition of calcifying VSMC-derived exosome production has been demonstrated (226). Prothrombin, like matrix Gla-protein, contains a Gla domain. Prothrombin’s Gla domain interacts with exosome surfaces, preventing calcification nucleation site formation on the exosomal surface (226). In addition, the miRNAs (miRNA99a/146b/378a) present in exosomes produced by bone marrow-derived macrophages polarized with IL-4 suppress inflammation and facilitate the polarization of recipient macrophages into M2 subtype (227). By infusing these exosomes repeatedly into *ApoE^–/–^* mice fed a Western diet, necrotic lesions are reduced (227). Collectively, this stabilizes atheroma, suggesting that cultured macrophage-produced exosomes have an anti-inflammatory effect (227). In addition, polarized macrophages are capable of resolving inflammation by releasing exosomes loaded with anti-inflammatory miRNAs, specifically in atherosclerotic lesions (227).

As a result, senescence-associated exosomes appear to be a specific SASP component that regulates the phenotype of target cells. Recent years have seen senescent cells used as therapeutic targets, with senomorphic drugs used to slow down SAPS activity and senolytic drugs intended to remove senescent cells. Essentially, these therapies exploit the fact that senescent cells exhibit a markedly enhanced SAPS activity, which, in atherosclerosis, modulates in an autocrine manner the activity of the senescent cell, as well as that of the cellular senescence process and associated damage in a paracrine and endocrine manner, related to epigenetic mechanisms. It is, however, important to note that these treatments that have proved effective in experimental models present challenges for their translation to clinical practice due to side effects that extend far beyond their senomorphic benefits. Instead, extracellular vesicles, such as exosomes, may serve as cell-free therapeutic agents. Not only were they not at risk of promoting tumourigenesis, but they were also less susceptible to immune rejection (228). An intriguing possibility is that exosomes may carry biological information which may be beneficial or detrimental, suggesting they can function as anti-aging agents (229).

The sources of therapeutic exosomes can be natural, modified, or artificial. There is a wide variety of substances contained in exosomes derived from stem cells that have antioxidant and anti-inflammatory properties, which ultimately contribute to anti-aging effects. In a study performed with exosomes derived from mesenchymal stem cells, oxidative damage, SASP expression, and proteins associated with aging, such as p53, were significantly reduced, resulting in a significant reduction in aging-related CD4^+^ T cell senescence (230). In this study, miR-21 was found to be responsible for this activity, which decreased phosphatase and tensin homolog (PTEN) and increased PI3K and AKT activation, subsequently inducing nuclear factor E2-related factor 2-induced antioxidant gene expression (230). Likewise, exosomes from mesenchymal stem cells reduced senescent biomarkers and SASP in oxidative stress-induced senescent ECs, with miR-146a acting as the mediator by downregulating Src activation and other downstream pathways (231). The high levels of RNase H2 in centenarians’ fibroblasts were accompanied by low levels of cytoplasmic RNA:DNA hybrids, a RNase H2 substrate, and indicators of pro-inflammatory responses, suggesting enhanced repair of highly frequent ribonucleotide DNA lesions (232). Therefore, anti-atherosclerosis *via* exosomes targeting substances related to SAPS may present new research opportunities. However, these attractive concepts have yet to be demonstrated *in vivo* and face a number of challenges: (1) to create animal models impaired in exosome generation in order to better understand the significance of exosomes and the cellular and molecular factors that induce their ambiguous involvement in the pathogenesis of atherosclerosis; (2) to characterize the exosome components of senescent cells, which contain a variety of substances such as miRNA, lipids, proteins, and other proinflammatory factors, ROS, or growth factors; (3) to further elucidate the exact cell of origin of exosomes since cellular senescence is a heterogeneous process that varies with the cell type and tissue and senescent cells may express distinct senescence markers, release different SASP factors, and exhibit diverse SCAPs to resist apoptosis depending on their origin (233); (4) to surmount technical hurdles such as exosome extraction and purification, as the development of exosomes for therapeutic treatment, drug administration, or theranostic applications necessitates high purity, low toxicity, and large-scale manufacturing; (5) to further validate in multicenter clinical cohorts the therapeutic efficacy of exosome manipulation.

It is possible that some emerging areas do not arouse scholarly interest immediately upon their appearance; as a result, detecting keywords with citation bursts alone may overlook these areas. Alternately, the timeline view (Figure 7) displaying the dynamic time change of co-occurring author keywords was employed to locate other frontiers in this field. The detrimental consequences of PM2.5 and its association with atherosclerosis, for instance, have received considerable attention in recent years.

Numerous epidemiological research on PM2.5 exposure and the health impacts of atherosclerosis have been undertaken. These studies all supported the association between PM2.5 and atherosclerosis, despite their use of cross-sectional and cohort epidemiological methods and subclinical indicators of atherosclerosis, such as carotid intimal-medial thickness, coronary artery calcium, thoracic aortic calcification, and ankle-brachial index (234–236). As concluded by these results, higher levels of PM2.5 (>10 μg/m^3^) are likely to represent a significant risk factor for developing subclinical atherosclerosis symptoms (234–236). Although PM2.5 levels at the threshold believed to be safe (within the annual World Health Organization Air Quality Guidelines <10 μg/m^3^), there is evidence that PM2.5 even at low concentrations (6.9 μg/m^3^) was associated with the development of atherosclerosis in asymptomatic adults with low cardiovascular risk, independent of other risk factors (237).

The close positive correlation between low levels of PM2.5 and cardiovascular mortality has also been demonstrated. The results of a cohort study involving 2.1 million Canadians showed significant increases in ischemic heart disease deaths with each 10 μg/m^3^ increase in PM2.5 levels, despite ambient PM2.5 levels averaging 8.7 μg/m^3^ on an annual basis (238). There are two other Canadian studies that indicate that long-term exposure can result in increased cardiovascular event risk at exposure levels as low as 6.3 and 5.9 μg/m^3^ (239, 240).

Atherosclerosis is influenced by several complex, multiple, and interdependent biological mechanisms caused by PM2.5. Essentially, PM2.5 promotes atherosclerosis through its systemic oxidative and inflammatory effects at large. There is a dose-effect relationship between PM2.5 concentration and the potential to trigger an inflammatory response and oxidative stress, which is positively connected with cardiovascular disease morbidity and mortality (141).

There is evidence that with increased PM2.5 exposure, various inflammatory markers and immune parameters associated with atherosclerosis are reported to be elevated, including high-sensitivity CRP, plasma fibrinogen, IL-6, immunoglobulin (Ig)G, IgM, and IgE (142, 241–243). As a result of inhalation of PM2.5, the respiratory epithelium can be damaged and local inflammation of the lung tissue can occur, which results in a wide variety of mediators released into the blood stream and initiates a systemic inflammatory response. Specifically, the NF-κB pathway is activated by PM2.5 when it binds to TLRs on lung macrophage surfaces, such as TLR2 or TLR4, which triggers inflammatory cytokine release (244). As a result of exposure of human alveolar macrophages to PM2.5, the production of the Th1 cytokines IL-12 and IFN-γ by M1 macrophages was increased, while the production of the Th2 cytokines IL-4, IL-10, and IL-13 by M2 macrophages was decreased (245, 246). In the M1 macrophages, inducible nitric oxide synthase expression was significantly increased after PM2.5 exposure, whereas CD20 and arginine-1 expression levels were significantly reduced in M2 macrophages (247). The polarization of M1 macrophages and the production of inflammatory cytokines are thus promoted by PM2.5. Other atherosclerosis-related pro-inflammatory cytokines, including granulocyte-macrophage colony-stimulating factor, IL-6, IL-1β, and TNF-α, were also elevated in M1 macrophages of C57BL/6 mice exposed to PM2.5 (248). Besides aggravating inflammation, PM2.5 exposure increases M1 macrophage numbers, which may underlie the development of atherosclerosis.

In addition to inhaled particulate matter reaching the terminal bronchioles and entering the alveoli, producing an inflammatory reaction in the lung with the subsequent release of inflammatory mediators into the circulation, a small fraction of particles also enter the circulation (249). In plaque macrophages, PM2.5 increases expression of CD36 and facilitates the abnormal accumulation of oxidized lipids (such as 7-ketocholesterol), finally resulting in the formation of foam cells (250). The TLR4/MyD88/NF-κB pathway can also be implicated in the formation of foam cells caused by PM2.5 (251). As a result of oxLDL binding to TLR4 or CD36, the NLRP3 inflammasome is primed and activated, causing the release of IL-1β and IL-18 and the activation of pyroptosis in macrophages. Additionally, PM2.5 induces the expression of cyclooxygenase-2 (COX-2) and microsomal prostaglandin E synthase-1 (mPGES-1) in vascular ECs in a concentration-dependent manner (252). The inhibition of COX-2 significantly reduced PM2.5-induced prostaglandin 2 (PGE2) production and attenuated the inflammatory response, suggesting that the COX-2/PGES/PGE2 pathway may play a role in atherosclerosis and vascular inflammation caused by PM2.5 (252). It has also been demonstrated that PM2.5 triggers the production of IL-6 and IL- IL-1β in HUVECs through activation of the TLR-mediated pathway, and that TLR2 and TLR4 inhibitors reduce the inflammatory response induced by PM2.5 (253). The effect of PM2.5 has been demonstrated to activate ECs, increase the expression of adhesion molecules (ICAM1 and VCAM-1) and promote the adhesion of THP-1 cells to endothelial cells through the extracellular-signal-regulated kinase (ERK)/AKT/NF-κB pathway in EA.hy926 cells; additionally, these effects have been demonstrated through the use of ERK/AKT/NF-κB inhibitors (254).

Further, particulate matter may trigger the development of atherosclerosis by causing oxidative stress. The heavy metals present on the surface of PM2.5 can catalyze the Fenton reaction to produce ROS whose high concentrations lead to the induction of NADPH oxidase, which causes mitochondrial damage (255, 256). In a recent study, PM2.5 was shown to damage mitochondria in macrophages, activate the mitochondria-mediated apoptosis pathway, increase lipid accumulation in macrophage foam cells, and aggravate atherosclerosis progression (257). Furthermore, PM2.5-mediated ROS can indirectly promote inflammation as well. Overexpression of ROS plays a major role in activating the NLRP3 inflammasome, which in turn regulates the expression of caspase-1, IL-18, and IL-1β (258, 259). The activation of NLRP3 inflammasomes was associated with the polarization of macrophages and the infiltration of M1 macrophages, which are responsible for the release of inflammatory cytokines (260, 261). Moreover, PM2.5 derived from cooking oil fumes also promotes autophagy in HUVECs through ROS/AKT/mTOR signaling (262).

Despite the prevailing view that PM2.5 is linked to atherosclerosis by activating inflammatory pathways and causing oxidative stress, epidemiological and clinical data indicate that PM2.5 can accelerate lipid buildup by modifying lipid metabolism and lipoprotein properties, particularly by promoting LDL oxidation and interfering with the function of scavenger receptors and LDL receptor (263). High-density lipoprotein (HDL) can reverse the transport of excess cholesterol in cells and extracellular tissues, breakdown physiologically active oxidative phospholipids in serum, and block the oxidation of LDL (264–266). The multiethnic cross-sectional study (MESA) based on atherogenic air pollution showed that HDL particle concentration decreased by 0.64 μmol/L (95% CI, −0.82 to 0.71) for every 5 μg/m^3^ increase in PM2.5 (267). Moreover, another study found that brief exposure to concentrated PM2.5 had an acute effect on HDL’s antioxidant and anti-inflammatory capacities (268). PM2.5 exposed mice exhibited a decrease in HDL cholesterols and apolipoprotein A1, along with an increase in ApoB, low-density LDL-C, and oxLDL, which is associated with the joint regulation of immune activation by CD36 and NLRP3, as compared to mice exposed to filtered air (269). The literature on PM2.5 exposure and HDL functionality also indicates that particulate matter may impair HDL functionality by oxidative mechanisms (270).

An additional PM2.5-related mechanism implicated in pathways leading to atherosclerosis is endothelial dysfunction. Air pollution-mediated cardiovascular diseases may be initiated by changes in vascular function, and indeed, these changes are a key early indicator of atherosclerosis (271, 272). EC dysfunction caused by PM2.5 is primarily a result of indirect cytotoxic effects caused by inflammation and oxidative stress.

Human lung endothelium was damaged by PM2.5 due to ROS generation resulting in disruption of the EC barrier and the release of a large number of cytokines *via* pathways dependent upon p38 mitogen-activated protein kinase and HSP27 (272, 273).

There have been a great number of studies conducted in recent years which demonstrate that PM2.5 can directly damage HUVECs. EA.hy926 cells and human HUVECs are affected by PM2.5 by reducing the mitochondrial membrane potential, increasing the generation of ROS, inducing oxidative stress, inflammation, and apoptosis (274–276).

Human umbilical vein endothelial cells and EA.hy926 cells are induced to undergo autophagy and apoptosis by PM2.5 *via* endoplasmic reticulum stress (277). Although autophagy appears to protect cells from PM2.5-induced apoptosis, PM2.5 blocks autophagic flux and further aggravates apoptosis in ECs (277–279). When endoplasmic reticulum stress is effectively inhibited by 4-PBA (an ER stress inhibitor), PM2.5-induced cell apoptosis and LC3II expression are alleviated (277). Transcellular permeability of vascular endothelial monolayers increases when excessive apoptosis occurs.

Iron uptake and storage are disrupted by ambient PM2.5 by regulating the expression of transferrin receptor, ferritin light chain and heavy chain, resulting in intracellular iron overload, which in turn triggers ferroptosis in EA.hy926 cells and HUVECs (280).

A further effect of PM2.5 is to activate senescence associated-β galactosidase in premature coronary ECs *via* redox sensitivity of the local angiotensin system, resulting in senescence of these cells (281). By introducing senescent cells into a non-senescent monolayer, the morphology of tight junction of surrounding young cells is disrupted and the monolayer’s permeability is increased (147).

The vascular endothelial-cadherin is predominantly expressed on endothelial cell membranes, where it is essential for maintaining the integrity of the endothelial barrier as well as controlling the movement of macromolecules across the membrane, such as blood cells (282). In response to PM2.5, vascular endothelial growth factor receptor 2 is phosphorylated and downstream signaling involving mitogen-activated protein kinase and ERK is activated, leading to the loss of adherens junction protein vascular endothelial-cadherin (283).

After vascular injury, PM2.5 exposure also impairs EC proliferation and migration, as well as promoting apoptosis and inhibiting angiogenesis (284). PM2.5 derived from cooking oil fumes can significantly reduce cellular viability and inhibit angiogenesis in HUVECs *via* the ROS-mediated NLRP3 inflammasome pathway or the vascular endothelial growth factor/vascular endothelial growth factor receptor 2/mitogen-activated protein kinase 1/2/ERK1/2/mTOR pathway (285).

In conclusion, the evidence suggests that PM2.5 increases endothelial cellular apoptosis *via* oxidative stress or autophagy, reduces the migration of ECs, and increases the permeability of vascular endothelium, thereby triggering a series of reactions in the development of atherosclerosis.

From both an epidemiological and experimental perspective, PM2.5 exposure is positively associated with atherosclerosis. There are several primary mechanisms involved, including inflammation, oxidative stress, abnormal lipid metabolism, and endothelial dysfunction.

It is important to note that the components of PM2.5 are complex and vary greatly in composition and concentration over time and space. However, there is limited knowledge of the critical pathogenic effects caused by specific PM2.5 components. The further investigation of the toxicity of PM2.5 components may contribute to an improved understanding of PM2.5, which may be one of the key areas of future study. There are also very few studies that have attempted to examine preventive measures. Molecule-level changes occur much earlier than histopathology and clinical symptoms. Thus, improved understanding of molecular mechanisms would make it possible to examine potential measures or targets that can contribute to the prevention of PM2.5-induced atherosclerosis, which remains a challenge until the environmental conditions are improved.

## Conclusion

This bibliometric profile of the literature specifically concerning atherosclerosis over the last decade aims to identify, evaluate and visualize research patterns in qualitative, quantitative, and chronological aspects. North American and European countries stood out in quantitative, qualitative, and collaborative parameters as a leading force in the field. To spur further academic research in Asia, it is determined that the degree of global cooperation needs to be improved. The current study suggests that research into vascular inflammation is a critical component of atherosclerosis studies. Targeting inflammation as an avenue to prevent CVDs has become a hot topic. An assessment of the literature found that the NLRP3 inflammasome and IL-1β, gut microbiota and SCFAs, exosomes, lncRNAs, autophagy, and cellular senescence were areas of major research foci.

## Data availability statement

The original contributions presented in this study are included in the article/Supplementary material, further inquiries can be directed to the corresponding authors.

## Ethics statement

Ethical review and approval was not required for this study in accordance with the local legislation and institutional requirements.

## Author contributions

HX and JJ designed this study. WT and TZ collected the data, normalized the pictures, and wrote the original draft. JZ and XW performed the analysis. HX, JJ, and TZ reviewed and revised the manuscript. All authors contributed to the article and approved the submitted version.
