# Supplementary material for: Global research trends in atherosclerosis: A bibliometric and visualized study
Source: Front Cardiovasc Med. 2022 Aug 23;9:956482. doi: 10.3389/fcvm.2022.956482 (PMC9445883; doi:10.3389/fcvm.2022.956482)
Supplement: Supplementary file 1 [file Data_Sheet_1.pdf]

Supplementary Material

1 Supplementary Figures and Tables

1.1 Supplementary Figures

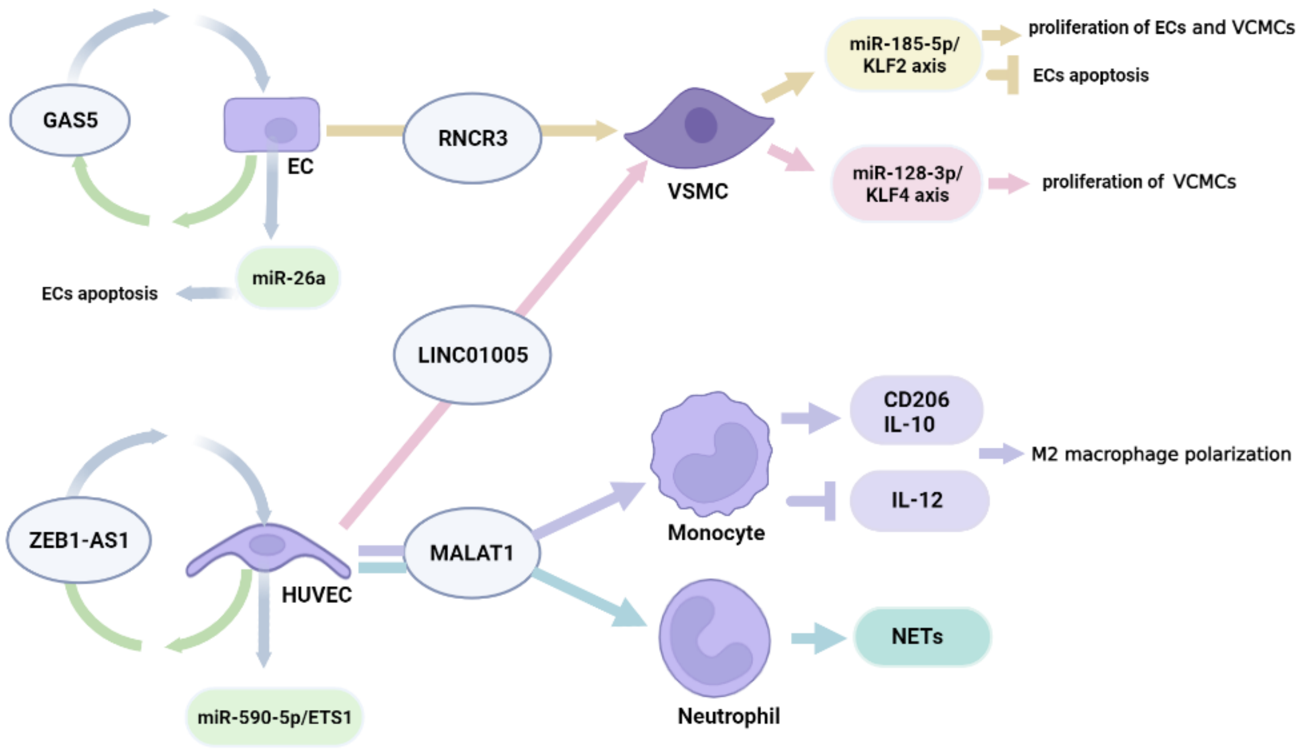

Supplementary Figure 1 Function of exosomal lncRNAs in atherosclerosis.

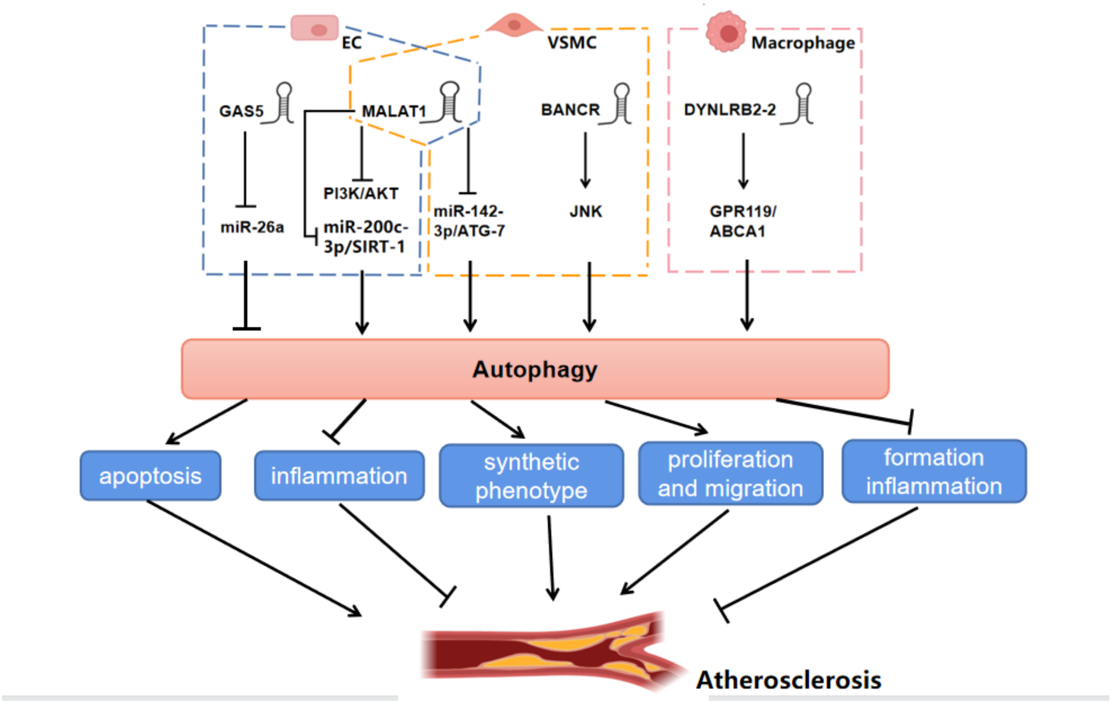

Supplementary Figure 2 LncRNA-mediated autophagy in atherogenesis.

1.2 Supplementary Tables

Supplementary Table 1 Overview of references with ongoing strongest citation bursts

Reference	Title	Key Findings or Conclusions
[43]	Antiinflammatory Therapy with Canakinumab for Atherosclerotic Disease	In anti-inflammatory, anti-thrombotic outcomes research on canakinumab, evidence has been presented that the IL-1 signaling pathway contributes to atherosclerosis. There were less recurrent events such as cardiovascular death, MI, and ischemic stroke after canakinumab, an anti-IL-1β antibody (300 mg four times per year).
[46]	Endothelial Cell Dysfunction and the Pathobiology of Atherosclerosis	During their study of endothelial cell dysfunction, the authors concluded that endothelial dysfunction plays a key role in the pathogenesis of arterial vascular system disease. They reviewed the evolution of concepts regarding endothelial dysfunction, particularly discussing the cellular and molecular mechanisms responsible for atherosclerosis.
[47]	Heart Disease and Stroke Statistics-2019 Update: A	Prepared by the American Heart Association each year in collaboration with the National Institutes of Health and other agencies, this report provides the most comprehensive data on cardiovascular disease statistics on a national level in the USA. As

	Report From the American Heart Association	reported, all major categories of cardiovascular disease were on the rise from 2014 to 2019. These include stroke, peripheral arterial disease, and coronary heart disease. Increases such as these highlight both the magnitude and ongoing progression of cardiovascular disease in the USA. Compared with traditional risk factor-based narratives, the report included a major focus on social determinants of health and explicitly discussed how it contributes to the overall burden of heart disease and stroke in the USA.
[48]	Atherosclerosis	This review provides an overview of atherosclerosis regarding epidemiology, pathogenesis in three phases: initiation, progression and complications, diagnosis, screening and prevention, and management. As this review identified, despite notable progress in understanding the mechanisms of atherosclerosis and the introduction of highly technological interventions in the treatment of complications that have resulted in survivors living longer, there are still issues including heart failure caused by ischaemic cardiomyopathy and poor quality of life. As a result, this raises awareness of some of the easier solutions to stemming the epidemic of atherosclerosis, which involve behavioural or societal changes. As our technological prowess has grown, our ability to adopt healthy dietary habits, regular physical activity, and tobacco cessation has not kept pace.
[49]	Vascular smooth muscle cells in atherosclerosis	In the occurrence and development of atherosclerosis, VSMCs play an indispensable role. As a result of altered gene expression, in pathological conditions the VSMCs transition from contractile (differentiated) to secretory (dedifferentiated) phenotypes, resulting in modifications in proliferation, migration, phagocytosis, and release of inflammation substances and collagen fibers, all of which may contribute to the formation and progression of atherosclerosis.
[50]	Inflammation and its resolution in atherosclerosis: mediators and therapeutic opportunities	A failure to resolve inflammation is what contributes to the development of atherosclerosis in all its stages, such as fatty streaks, fibrous plaques, ruptured acute plaques, and thrombi formation.
[51]	Immunity and Inflammation in Atherosclerosis	As a consequence of the importance of LDL in the initiation of atherosclerotic lesions along with the presence of auto-reactive T and B cells as well as anti-oxLDL antibodies, the idea of the autoimmune component of atherosclerosis has developed. There is a Th1-dominant aspect to atherosclerosis in that Th1 cells recognize antigens and release proinflammatory cytokines such as IL-2, interferon-γ (IFN-γ), and tumor necrosis factor-alpha. Th2 and Th17 have pro-atherogenic and anti-atherogenic effects. Regulatory T cells are protective of atherosclerosis.

[52]	Efficacy and Safety of Low-Dose Colchicine after Myocardial Infarction	Patients within 30 days of a MI randomized to colchicine 0.5 mg daily had reduced primary (cardiovascular mortality, resuscitated cardiac arrest, MI, stroke, or urgent hospitalization for angina leading to coronary revascularization) and secondary (cardiovascular mortality, all-cause mortality, MI, stroke, and resuscitated cardiac arrest) end points significantly by 23% and 15%, respectively compared to placebo-treated patients in The Colchicine Cardiovascular Outcomes Trial.
[53]	Heart Disease and Stroke Statistics-2020 Update: A Report From the American Heart Association	Even with significant improvements in treatment options, cardiovascular disease affects 48% of all adults older than 20 years of age, incurring costs in excess of 350 billion dollars every year, causing nearly 860,000 deaths in the USA and 17.8 million deaths worldwide.
[54]	2019 ESC/EAS Guidelines for the management of dyslipidaemias: lipid modification to reduce cardiovascular risk	The 2019 European Society of Cardiology/European Atherosclerosis Society (ESC/EAS) Guidelines for the Management of Dyslipidemias offers new recommendations for the management of blood lipid levels based on the latest evidence. In general, the recommendations are based on the well-established causal link between elevated levels of LDL cholesterol and ASCVD. Several of its highlights can be appreciated in this reference [72] which compares and contrasts the common features and differences between the 2019 ESC/EAS Guidelines and The 2018 American Heart Association/American College of Cardiology/Multi-Society (AHA/ACC/MS) Management of Blood Cholesterol.
